# Dynamic gold rush optimizer: fusing worker adaptation and salp navigation mechanism for enhanced search

**DOI:** 10.1038/s41598-025-00076-5

**Published:** 2025-05-06

**Authors:** Yanhua Zhang, Oluwatayomi Rereloluwa Adegboye, Afi Kekeli Feda, Ephraim Bonah Agyekum, Pankaj Kumar

**Affiliations:** 1 Department of Physics and Electronic Engineering, Yuncheng University, Yuncheng City, Shanxi Province China; 2 University of Mediterranean Karpasia, Mersin-10, Northern Cyprus, TR-10, Mersin, Turkey; 3 Advanced Research Centre, European University of Lefke, Northern Cyprus, TR-10, Mersin, Turkey; 4 Department of Nuclear and Renewable Energy, Ural Federal University Named after the First President of Russia Boris, 19 Mira Street, Ekaterinburg, Yeltsin, 620002 Russia; 5 Department of Science and Innovations, Western Caspian University, Baku, AZ1001 Azerbaijan; 6 Tashkent State University of Economics, Islam Karimov street 49, Tashkent City, 100066 Uzbekistan; 7 Department of Electrical and Electronics Engineering, Manipal Institute of Technology, Manipal Academy of Higher Education, Manipal, 576104 Karnataka India

**Keywords:** Gold rush optimizer, Numerical optimization, Salp navigation mechanism, Workers adaption strategy, Metaheuristic algorithm, Engineering, Electrical and electronic engineering

## Abstract

The Dynamic Gold Rush Optimizer (DGRO) is presented as an advanced variant of the original Gold Rush Optimizer (GRO), addressing its inherent limitations in exploration and exploitation. While GRO has demonstrated efficacy in solving optimization problems, its susceptibility to premature convergence and suboptimal solutions remains a critical challenge. To overcome these limitations, DGRO introduces two novel mechanisms: the Salp Navigation Mechanism (SNM) and the Worker Adaptation Mechanism (WAM). The SNM enhances both exploration and exploitation by dynamically guiding the population through a stochastic strategy that ensures effective navigation of the solution space. This mechanism also facilitates a smooth transition between exploration and exploitation, enabling the algorithm to maintain diversity during early iterations and refine solutions in later stages. Complementing this, the WAM strengthens the exploration phase by promoting localized interactions among individuals within the population, fostering adaptive learning of promising search regions. Together, these mechanisms significantly improve DGRO’s ability to converge toward global optima. A comprehensive experimental evaluation was conducted using benchmark functions from the Congress on Evolutionary Computation (CEC) CEC2013 and CEC2020 test suites across 30 and 50-dimensional spaces, alongside seven complex engineering optimization problems. Statistical analyses, including the Wilcoxon Rank-Sum Test (WRST) and Friedman Rank Test (FRT), validate DGRO’s superior performance, demonstrating significant advancements in optimization capability and stability. These findings underscore the effectiveness of DGRO as a competitive and robust optimization algorithm.

## Introduction

An optimization problem is defined as the process of minimizing or maximizing an objective function subject to a specific set of constraints. The primary goal in addressing an optimization problem is to identify the optimal solution that represents the most favourable outcome among all possible alternatives^1^. In practice, there is often a need to achieve the optimal solution for specific problems in a variety of circumstances and fields. One typical example is the travelling salesman problem, which requires determining the most efficient path for a salesman to visit several cities once and return to the starting position. By optimizing the route, the salesman aims to minimize the total distance travelled and, consequently, reduce costs and save time^2^. Optimization problems are encountered in numerous applications. Structural optimization^3^, spacecraft trajectory optimization^4^, neural network optimization^5^, predictive modelling^6^, photovoltaic system design^7^, precision optical equipment development^8^, or allocation problems^9^. With time, the complexity of these problems has increased to surpass the capabilities of traditional methods to solve them^10^. In response, researchers have developed MAs, which offer improved and faster solutions to complex optimization problems by imitating social behavior, physics laws, or natural phenomena^11^. MAs have considerable advantages^12^. Firstly, their inherent randomness enables the avoidance of local extrema and facilitates comprehensive exploration of the search space. Secondly, their black-box design allows MAs to operate solely based on the input and output of a problem without requiring gradient information. Additionally, these algorithms rely on simple mathematical models, making them straightforward to implement^13^. Recent MAs inspired by either of these three phenomena include: Artificial Rabbit Optimization (ARO) is inspired by the resilience tactics of naturally bilaterally symmetric rabbits^13^. Fire Hawk Optimizer (FHO) motivated by the hunting patterns of black kites, brown falcons, and whistling kites^14^, Honey Badger Algorithm (HBA) based on the sophisticated hunting habits of honey badgers^15^; the Snow ablation optimizer simulating the sublimation and melting characteristics of snows^16^; Gannet optimization algorithm (GOA) which imitates the different distinctive actions that gannets take when foraging^17^, and the Dandelion Optimizer (DO) that replicates the wind-powered flight of dandelion seeds^18^.

Despite their many advantages, metaheuristic algorithms suffer from some limitations, such as the difficulty in balancing the exploration for new solutions and the exploitation of existing ones. Most MAs also suffer from premature convergence and low population diversity, which can lead to local stagnation and sub-optimum solutions. Hybrid algorithms have emerged as a promising trend in the field of optimization for tackling these problems. These methods combine key features from different MAs and optimization techniques, aiming to leverage their individual strengths. As a result, hybrid algorithms are better equipped to explore the solution space thoroughly while maintaining focused exploitation. Their ability to strike this balance often leads to faster convergence, higher solution quality, and greater overall efficiency^19^. Recent examples of hybrid algorithms include the Reptile Search Algorithm combined with Salp Swarm Algorithm(RSA-SSA)^20^. The enhanced slime mould algorithm based on an adaptive grouping technique^21^. The Hybrid Algorithm of Differential Evolution and Flower Pollination (HADEFP)^22^. The improved real-coded genetic algorithm (RCGA-rdn) including three specially designed operators: ranking group selection (RGS), direction-based crossover (DBX), and normal mutation (NM)^23^. The improved chaos Sparow search algorithm through chaotic initialization, dynamic weighting, expanded search via an enhanced sine cosine update, and reverse learning^24^. The Hybrid Grasshopper Optimization Algorithm (HGOA) merges an improved Grasshopper Optimization Algorithm featuring a nonlinear control parameter with a modified Butterfly Optimization Algorithm that employs dynamic inertia weights using a probabilistic selection strategy for integration^25^. The improved dung beetle optimization algorithm (MDBO) uses Latin hypercube sampling, Mean Differential Variation, a hybrid reverse learning and dimension-wise optimization strategy^26^, and The reinforced JAYA algorithm (QLJAYA) that employs the Q-learning and gradient search scheme^27^.

The Gold Rush Optimizer (GRO) is a recently developed MA inspired by the three fundamental strategies employed by gold prospectors during the gold search: migration, gold mining, and collaboration^28^. GRO has gained popularity for optimization tasks due to its strong global search capacity, leading to effective exploration of the solution space. Furthermore, GRO possesses straightforward implementation steps and few parameters. These properties make the GRO a competitive and appealing choice among the several metaheuristic algorithms accessible in the literature. While GRO has demonstrated significant potential in solving optimization problems^29–31^, like other MAs it suffers from notable limitations. Specifically, the algorithm converges prematurely, thereby reducing its effectiveness in exploring the broader search space. This premature convergence results in the algorithm becoming trapped in local optima, thus hindering its ability to identify the global optimum due to its inherently limited exploration mechanism. A few researchers have proposed solutions and introduced enhanced versions of the GRO to address its limitations and improve its performance in tackling premature convergence, escaping local optima, and lack of broad exploration capacity. Lyu et al. proposed the Augmented Gold Rush Optimizer (AGRO), incorporating multiple mechanisms to enhance its performance. These mechanisms include point-set population initialization, Lévy Flight Search (LFS), dynamic centroid reverse learning, and dynamic tangent flight. Experimental results demonstrated that AGRO exhibits high scalability and improved performance, making it a robust approach for addressing complex optimization problems^32^. Kong et al. improved the migration strategy of the GRO by integrating the LFS and introducing dynamic opposition learning to enhance population diversity. Additionally, a multi-player collaborative strategy was employed to improve the algorithm’s search ability during the collaboration phase, while random differential mutation was utilized to avoid local optima. The experimental results showed significant improvements in convergence and overall performance^33^. Nie et al. introduced logistic-tent mapping to develop the Chaotic Gold Rush Optimizer (CGRO), addressing the challenge of premature convergence to local optima. This enhancement facilitated the design of linear antenna arrays with improved directivity and reduced side lobe levels (SLL). Findings demonstrated the improved performance and applicability of CGRO in solving complex engineering optimization problems^34^.

Despite various attempts in the literature to enhance the performance of the GRO, challenges persist due to its limited exploration capabilities, which often result in premature convergence and stagnation in local optima. These shortcomings stem from GRO’s inability to maintain an optimal balance between exploration and exploitation, particularly in high-dimensional and multimodal optimization landscapes. To address these deficiencies, this study introduces a novel variant, the Dynamic Gold Rush Optimizer (DGRO), which integrates two innovative mechanisms: the Salp Navigation Mechanism (SNM) and the Worker Adaptation Mechanism (WAM). The SNM is designed to enhance both exploration and exploitation by introducing adaptive and dynamic movements that guide the search process effectively. This mechanism mitigates the risk of stagnation by incorporating stochastic behavior, enabling the algorithm to escape local optima and explore diverse regions of the solution space. Furthermore, the SNM facilitates a seamless transition between exploration and exploitation, ensuring that the algorithm focuses on broad exploration during early iterations while progressively refining solutions in later stages.

Complementing the SNM, the WAM strengthens the exploration phase by fostering localized interactions and cooperative behaviors among individuals within the population. This mechanism leverages peer-driven interactions to enhance the neighborhood search process, enabling gold prospectors to iteratively refine solutions through adaptive learning. By concentrating the search on promising regions of the solution space, the WAM accelerates convergence while maintaining the algorithm’s capacity to identify global optima. Collectively, these mechanisms address the limitations of the original GRO, establishing DGRO as a highly effective and competitive optimization algorithm capable of tackling complex optimization problems with improved robustness and efficiency.

The remainder of this work is organized as follows: Sect. 2 provides a detailed description of the original GRO algorithm. Section 3 introduces the proposed DGRO, highlighting its novel modifications. Section 4, the experimental section, presents a comprehensive evaluation of DGRO’s performance using CEC benchmark functions and engineering problems, comparing it to other algorithms to validate its effectiveness. Finally, Sect. 5 concludes the study and outlines potential directions for future research.

## Original GRO

The GRO is a population-based MA inspired by the historical behaviors of gold prospectors during the gold rush. It combines three core concepts migration, gold mining, and collaboration, to balance exploration and exploitation in the search space. The algorithm initializes a population of gold prospectors with random positions in a $\:d$-dimensional search space. Each gold prospector’s position is stored in a matrix MGP as seen Eq. (1)

1 $$\:MGP=\left[\begin{array}{cccc}{x}_{11}&\:{x}_{12}&\:\cdots\:&\:{x}_{1d}\\\:{x}_{21}&\:{x}_{22}&\:\cdots\:&\:{x}_{2d}\\\:\vdots&\:\vdots&\:\ddots\:&\:\vdots\ \\{x}_{n1}&\:{x}_{n2}&\:\cdots\:&\:{x}_{nd}\end{array}\right]$$

where $\:n$ is the number of gold prospectors and $\:{x}_{ij}$ represents the $\:j$-th dimensional position of the $\:i$-th gold prospector. The fitness of each gold prospector is evaluated using an objective function $\:f$, with the solution stored in an evaluation matrix $\:MF$ in Eq. (2)

2 $$\:MF=\left[\begin{array}{c}f\left({x}_{11},{x}_{12},\dots\:,{x}_{1d}\right)\\\:f\left({x}_{21},{x}_{22},\dots\:,{x}_{2d}\right)\\\:\vdots\\\:f\left({x}_{n1},{x}_{n2},\dots\:,{x}_{nd}\right)\end{array}\right]$$

The position of the best gold prospector at each iteration is denoted by $\:{\overrightarrow{X}}^{\text{*}}$. Migration represents the movement of gold prospectors toward the most optimal known gold mine, and this is represented by the gold prospector with the most optimal solution. The position of a gold prospector $\:i$ is updated using Eqs. (3) and (4).

3 $$\:{\overrightarrow{D}}_{1}={\overrightarrow{C}}_{1}\cdot\:\left({\overrightarrow{X}}^{\text{*}}\left(t\right)-{\overrightarrow{X}}_{i}\left(t\right)\right)$$

4 $$\:{\overrightarrow{X}}_{i}^{\text{new\:}}(t+1)={\overrightarrow{X}}_{i}\left(t\right)+{\overrightarrow{A}}_{1}\cdot\:{\overrightarrow{D}}_{1}$$

Where $\:{\overrightarrow{X}}_{i}\left(t\right)$ denote the current position of gold prospector $\:i$, $\:{\overrightarrow{X}}^{\text{*}}\left(t\right)$ represent the position of the best gold prospector, $\:{\overrightarrow{A}}_{1}$ and $\:{\overrightarrow{C}}_{1}$ represent coefficients, which are computed as expressed in Eqs. (5) and (6)

5 $$\:{\overrightarrow{A}}_{1}=1+{l}_{1}\cdot\:\left({\overrightarrow{r}}_{1}-0.5\right)$$

6 $$\:{\overrightarrow{C}}_{1}=2\cdot\:{\overrightarrow{r}}_{2}$$

Where $\:{\overrightarrow{r}}_{1}$ and $\:{\overrightarrow{r}}_{2}$ are random vectors in the range $\:\left[\text{0,1}\right]$, $\:{l}_{1}$ is a convergence control factor defined in Eq. (7). represents the behavior of a nonlinear decay function if the parameter is set to a value above 1 and it decays linearly from 2 to $\:\frac{1}{\text{\:maxiter\:}}$ when its value is 1. $\:t$ represents the current iteration, and $\:\text{maxiter}$ represents the maximum number of iterations.

7 $$\:{l}_{1}={\left(\frac{\text{\:maxiter\:}-t}{\text{\:maxiter\:}-1}\right)}^{e}\cdot\:\left(2-\frac{1}{\text{\:maxiter\:}}\right)+\frac{1}{\text{\:maxiter\:}}$$

The gold mining concept simulates gold prospectors searching for gold around their current positions. A gold prospector $\:{\overrightarrow{X}}_{i}$ selects another gold prospector $\:{\overrightarrow{X}}_{r}$ randomly and updates its position using Eqs. (8) and (9)

8 $$\:{\overrightarrow{D}}_{2}={\overrightarrow{X}}_{i}\left(t\right)-{\overrightarrow{X}}_{r}\left(t\right)$$

9 $$\:{\overrightarrow{X}}_{i}^{\text{new\:}}(t+1)={\overrightarrow{X}}_{r}\left(t\right)+{\overrightarrow{A}}_{2}\cdot\:{\overrightarrow{D}}_{2}$$

Where $\:{\overrightarrow{A}}_{2}$ is calculated using Eq. (10)

10 $$\:{\overrightarrow{A}}_{2}=2\cdot\:{l}_{2}\cdot\:{\overrightarrow{r}}_{1}-{l}_{2}$$

The $\:{l}_{2}$ present an exploitation control parameter. The parameter adjusts the intensity and scope of local search, therefore $\:{l}_{2}$ is used instead of $\:{l}_{1}$. The collaboration concept models teamwork among gold prospectors. Two random gold prospectors $\:{\overrightarrow{X}}_{{g}_{1}}$ and $\:{\overrightarrow{X}}_{{g}_{2}}$ define the search region, and the new position of gold prospector $\:{\overrightarrow{X}}_{i}$ is calculated using Eqs. (11) and (12).

11 $$\:{\overrightarrow{D}}_{3}={\overrightarrow{X}}_{{g}_{2}}\left(t\right)-{\overrightarrow{X}}_{{g}_{1}}\left(t\right)$$

12 $$\:{\overrightarrow{X}}_{i}^{\text{new\:}}(t+1)={\overrightarrow{X}}_{i}\left(t\right)+{\overrightarrow{r}}_{1}\cdot\:{\overrightarrow{D}}_{3}$$

The $\:{\overrightarrow{r}}_{1}$ the parameter represents a random vector within $\:\left[\text{0,1}\right]$. Each gold prospector updates its position based on the fitness improvement as expressed in Eq. (13). The pseudocode of GRO is detailed in algorithm 1.

13 $$\:{\overrightarrow{X}}_{i}(t+1)=\left\{\begin{array}{ll}{\overrightarrow{X}}_{i}^{\text{new\:}}(t+1),&\:\text{\:if\:}f\left({\overrightarrow{X}}_{i}^{\text{new\:}}\right)<f\left({\overrightarrow{X}}_{i}\right)\\\:{\overrightarrow{X}}_{i}\left(t\right),&\:\text{\:otherwise\:}\end{array}\right.$$

**Algorithm 1:**
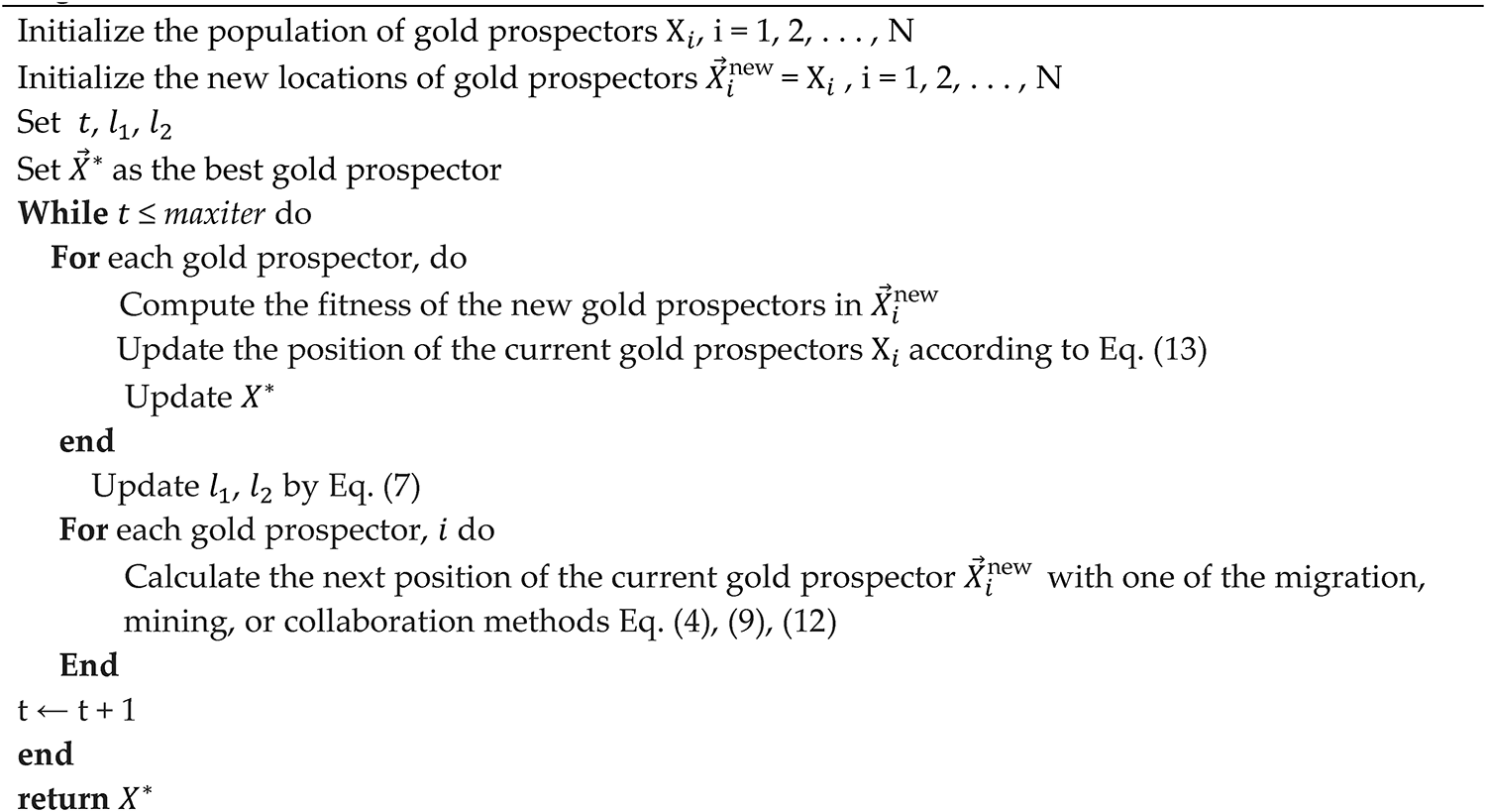
GRO Pseudocode.

## Proposed DGRO

### Salp navigation mechanism (SNM)

The Salp Swarm Algorithm (SSA) draws inspiration from the collective foraging behaviors of salps, marine creatures that navigate isn synchronized chains to locate food sources^35^. In SSA, the population of salps is mathematically modeled as a swarm, with a designated leader guiding the population of salps, referred to as followers. The leader’s navigation mechanism, critical for exploration and exploitation, is defined by Eq. (14)

14 $$\:{\overrightarrow{X}}_{i}^{\text{new\:}}(t+1)=\left\{\begin{array}{ll}{\overrightarrow{X}}^{\text{*}}+{c}_{1}*\left(\left({ub}_{i}-{lb}_{i}\right)\cdot\:{r}_{3}+{lb}_{i}\right),&\:{r}_{4}<0.5\\\:{\overrightarrow{X}}^{\text{*}}-{c}_{1}*\left(\left({ub}_{i}-{lb}_{i}\right)\cdot\:{r}_{3}+{lb}_{i}\right),&\:{r}_{4}\ge\:0.5\end{array}\right.$$

Where, $\:{\overrightarrow{X}}^{\text{*}}$ is food source position, which is the global optimal position found so far in the $\:i$-th dimension. $\:{ub}_{i},{lb}_{i}$ denote upper and lower bounds of the $\:i$-th dimension, respectively.$\:\:{r}_{3}$ is a uniformly distributed random value in $\:\left[\text{0,1}\right]$. $\:\:{r}_{4}$ is random is a variable in $\:\left[\text{0,1}\right]$, determining the movement direction toward or away from the food source. This exponential function decreases the value of $\:{c}_{1}.$ As the algorithm progresses, the search behavior of the salp population is dynamically controlled. The parameter $\:{c}_{1}\:$in the Eq. (14) plays a pivotal role in balancing exploration and exploitation during the optimization process. It is defined in Eq. (15).

15 $$\:{c}_{1}={\text{e}}^{-\left(\frac{4t}{\text{maxiter}}\right)2}$$

Where $\:t$ and $\:\text{maxiter}$ represent the current and maximum number of iterations. At the beginning of the optimization process ($\:t$ close to 0), $\:{c}_{1}$ has a relatively large value due to the exponential function. This amplifies the influence of the stochastic term $\:\left(\left({\text{u}\text{b}}_{i}-{\text{l}\text{b}}_{i}\right)\cdot\:{r}_{3}+{\text{l}\text{b}}_{i}\right)$ in the SNM update of DGRO’s gold prospectors as seen in Eq. (14). As the number of iterations increases ($\:t$ approaches $\:\text{maxiter}$), $\:{c}_{1}$ decreases exponentially. This reduction narrows the influence of the stochastic term, leading to smaller step sizes. This focuses on gold prospector movements around the location of the best gold mine ($\:{\overrightarrow{X}}^{\text{*}}$), emphasizing exploitation. Due to this behavior the algorithm performs fine-tuning to refine the best solutions found. It ensures convergence toward the global optimum. A dynamic balance between exploration and exploitation is introduced through the exponential behaviour of $\:{c}_{1}$ it ensures a smooth transition from exploration to exploitation. The new navigation mechanism is incorporated to refine the migration and collaboration phases of GRO. The SNM is utilized to adaptively guide GRO’s exploration of the search space, local optimal avoidance, improvement in population diversity, and the ability to transition between exploration and exploitation seamlessly.

### Worker adaptation mechanism (WAM)

The Worker Phase in the Naked Mole Rat Algorithm (NMRA) models the optimization process of workers striving to improve their status to breeders^36^. This mechanism can be adapted to enhance the GRO by integrating a structured exploration phase inspired by worker interactions. In the Worker Phase, each worker updates its position based on its own experience and local interactions. The update mechanism is defined mathematically in Eq. (16)

16 $$\:{\overrightarrow{X}}_{i}^{\text{new\:}}(t+1)={\overrightarrow{X}}_{i}^{\text{}}\left(t\right)+k\cdot\:\left({\overrightarrow{X}}_{j}^{\text{}}\left(t\right)-{\overrightarrow{X}}_{k}^{\text{}}\left(t\right)\right)$$

Where $\:{\overrightarrow{X}}_{i}^{\text{}}\left(t\right)$ denote the position of the $\:i$-th worker at iteration $\:t$. $\:{\overrightarrow{X}}_{i}^{\text{new\:}}(t+1)$ represents the updated position of the $\:i$-th worker at iteration $\:t+1$. $\:{\overrightarrow{X}}_{j}^{\text{}}\left(t\right),{\overrightarrow{X}}_{k}^{\text{}}\left(t\right)$ represent positions of two randomly selected workers at iteration $\:t$. $\:k$ is a random factor drawn from a uniform distribution $\:\left[\text{0,1}\right]$, regulating the step size. This equation ensures that workers leverage information from peers to explore new positions, thereby increasing diversity and enhancing the local search process. The adaptation of WAM into GRO, contributes to the algorithm’s gold mining phase by introducing structured randomness. Gold prospectors in GRO update their positions through improved peer interaction, as represented in Eq. (16). The integration of the WAM offers several significant improvements. It facilitates the use of peer information to enable gold prospectors to explore their neighborhoods effectively, mimics adaptive learning to enhance the algorithm’s ability to search for optimal solutions during the search process, and promotes interactions.

**Algorithm 2:**
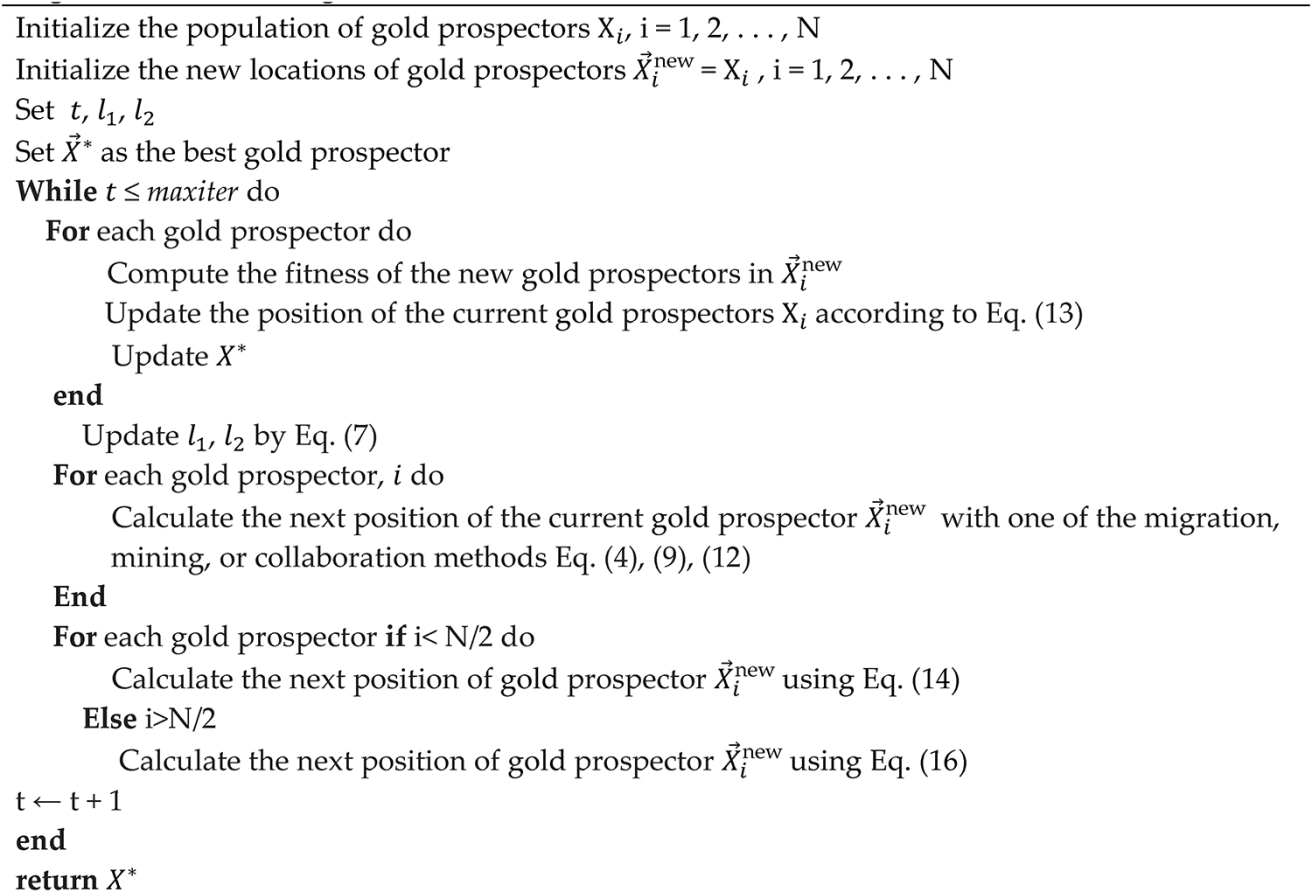
DGRO algorithm.

**Fig. 1:**
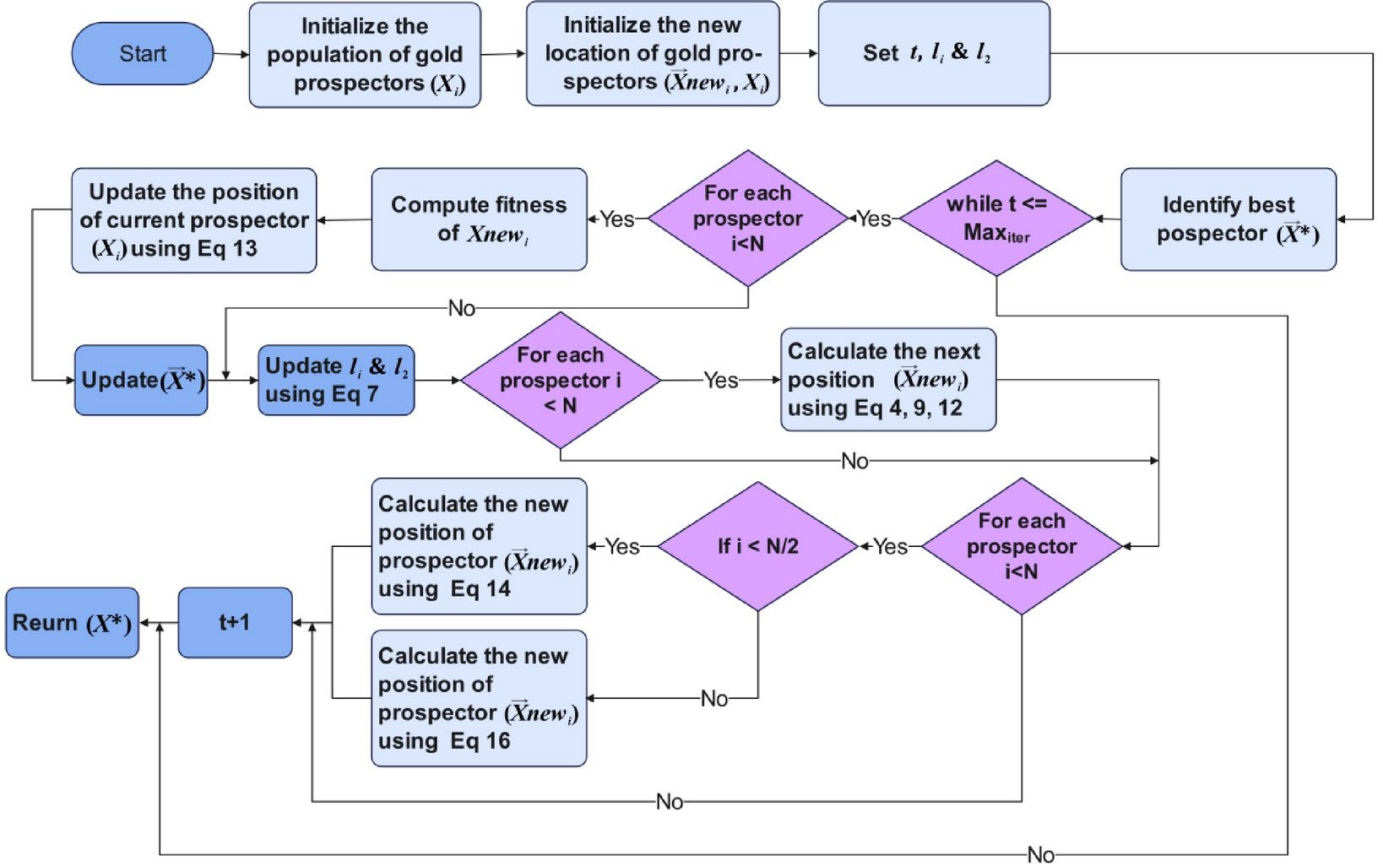
DGRO Flow Chart.

The presented pseudocode in Algorithm 2 outlines the procedural flow of the DGRO, and how it incorporates the SNM and the WAM to enhance exploration, exploitation, and convergence efficiency. The algorithm begins by initializing the population of gold prospectors, the global best gold prospector $\:{\overrightarrow{X}}^{\text{*}}$ is identified and updated iteratively throughout the process. During each iteration, the algorithm evaluates the fitness of the new gold prospectors and updates their positions based on Eq. (13), ensuring an improved population state. The parameters $\:{l}_{1}$ and $\:{l}_{2}$ are dynamically updated using Eq. (7) to balance the exploration and exploitation phases. Each gold prospector’s next position is calculated through one of the migration, mining, or collaboration methods by utilizing Eqs. (4), (9), or (12). For improved adaptability, the DGRO algorithm incorporates the SNM in Eq. (14) for the first half of the gold prospectors ($\:i<N/2$), guiding exploration, avoiding local optima, and enhancing population diversity. The second half of the prospectors ($\:i>N/2$) utilizes the WAM in Eq. (16) to mimic peer-based adaptive learning, enabling interactions and accelerating convergence. This iterative process continues until the maximum number of iterations is reached ($\:t\le\:$ maxiter). The algorithm returns the globally optimized solution$\:\:\overrightarrow{X}$. The inclusion of these mechanisms ensures robust performance across diverse optimization landscapes. The flow chart of DGRO is illustrated in Fig. 1. The source of code DGRO is given at https://github.com/MetaHeuLab/DGRO.

### Computational complexity of DGRO

The time complexity of the DGRO is determined by the computational requirements of its initialization phase and iterative processes. During initialization, the algorithm initialises the population of $\:N$ gold prospectors and their positions in a $\:d$-dimensional search space, resulting in a complexity of $\:\mathcal{O}(N\cdot\:d)$. In the iterative phase, which runs for a maximum of $\:\text{maxiter}$ iterations, the algorithm performs several key operations. Fitness evaluation, which is applied to all $\:N$ gold prospectors, incurs a complexity of $\:\mathcal{O}(N\cdot\:f(d\left)\right)$, where $\:f\left(d\right)$ represents the cost of the fitness function. Position updates, encompassing migration, mining, collaboration, and the newly introduced SNM and WAM mechanisms, contribute a complexity of $\:(N\cdot\:d)$. Additionally, dynamic parameter updates for $\:{l}_{1}$ and $\:{l}_{2}$ require constant time, $\:\mathcal{O}\left(1\right)$, per iteration. Combining these components, the per-iteration complexity is $\:\mathcal{O}(N\cdot\:(f\left(d\right)+d)$), leading to an overall complexity of $\:\mathcal{O}\left(\text{maxiter}\cdot\:N\cdot\:\left(f\right(d)+d)\right)$. This indicates that the computational cost of DGRO scales linearly with the population size, dimensionality, and the number of iterations, as well as the complexity of the fitness function.

## Experiments results and discussion

In this study, the proposed optimization method, DGRO, is rigorously evaluated using a comprehensive set of 38 benchmark optimization functions. These functions are derived from the CEC2013^37^ and CEC2020^38^ test suites. The CEC2013 test suite encompasses three distinct categories: unimodal functions (F1–F5), multimodal functions (F6–F20), and composite functions (F21–F28). Similarly, the CEC2020 test suite includes unimodal functions (F29), multimodal functions (F30–F32), hybrid functions (F33–F35), and composition functions (F36–F38). The primary objective of these benchmark functions is to assess the performance and convergence behavior of the DGRO method across a diverse range of optimization scenarios. Given that optimization algorithms are inherently stochastic, an experimental approach involving 30 independent runs is adopted to account for the randomness and variability inherent in the results. The Average (AVG) and Standard Deviation (STD) of the objective function values are recorded. This multiple-run strategy ensures robustness in performance evaluation. The parameter configurations of each algorithm are provided in Table 1. The study benchmarks DGRO against several recently developed and enhanced MAs, including the original GRO, Grey Wolf Optimizer (GWO)^39^, Honey Badger Algorithm (HBA)^15^, Parrot Optimizer (PO)^40^, Sine Cosine Algorithm (SCA)^41^, Seagull Optimization Algorithm (SOA)^42^, Transient Search Algorithm (TSO)^43^, Opposition-Based Learning PathFinder Algorithm (OBLPFA)^44^, Random Walk Grey Wolf Optimizer (RWGWO)^45^, and Time-Based Leadership Salp-Based Algorithm with Competitive Learning (TBLSBCL)^46^. These algorithms provide a robust baseline for evaluating the competitiveness of DGRO in solving optimization problems. For all experiments, a population size of 30 and a maximum iteration count of 5000 were used. Additionally, to evaluate scalability, both test suites were executed on problem dimensions of 30 and 50. This thorough experimental setup ensures a comprehensive assessment of DGRO’s performance and its ability to address complex optimization challenges effectively.

**Table 1:** Parameter configuration.

Optimizer	Parameter Settings
DGRO	$\:{l}_{1}=1,\:{l}_{2}=2,\:{c}_{1}\in\:\left[\text{0,2}\right]$
GRO	$\:{l}_{1}=1,\:{l}_{2}=2$
GWO	$\:{a}_{0}=2$
HBA	$\:\beta\:=6$, C = 2
OBLPFA	$\:\alpha\:,\:\beta\:=[1,\:2]$
PO	$\:\alpha\:$ = rand[0,1]/5, $\:\theta\:$ = rand[0,1] *$\:\:\pi\:$
RWGWO	$\:{a}_{0}=2$
SCA	$\:a\:=2$
SOA	$\:\text{A}\:\in\:\left[\text{2,0}\right],\:{f}_{c}=2$
TBLSBCL	$\:\varphi\:$ = 0.3, c_1_ =$\:[2/e,2]$
TSO	$\:k=2,\:z\in\:\left[\text{0,2}\right]$

### Experimental results of CEC2013

**Fig. 2:**
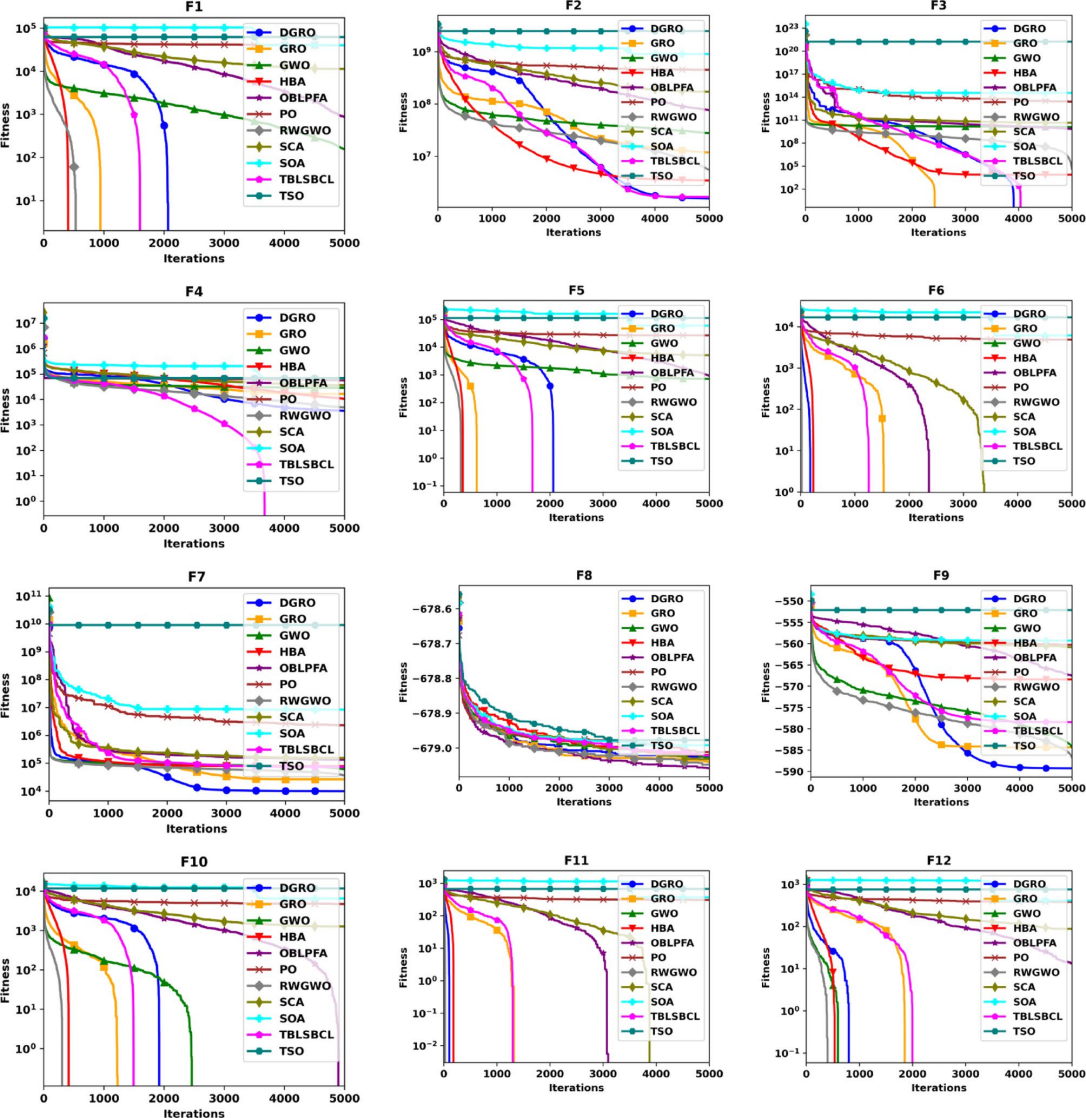

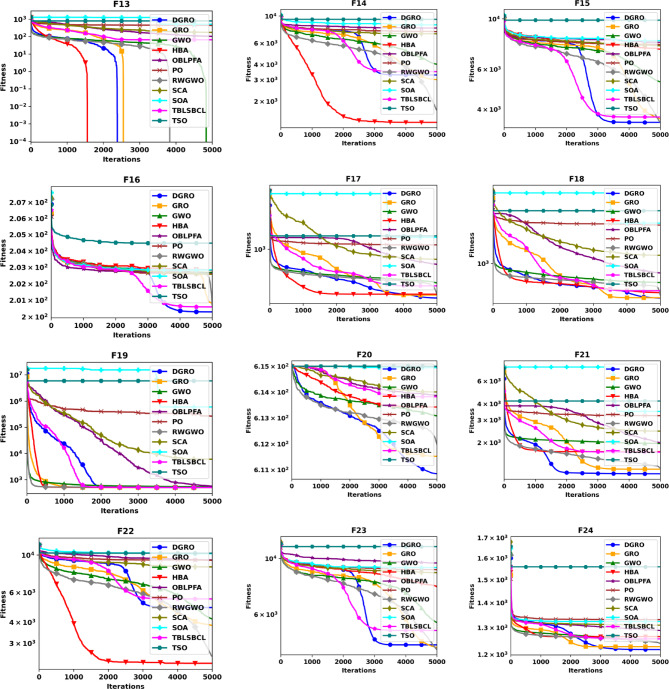

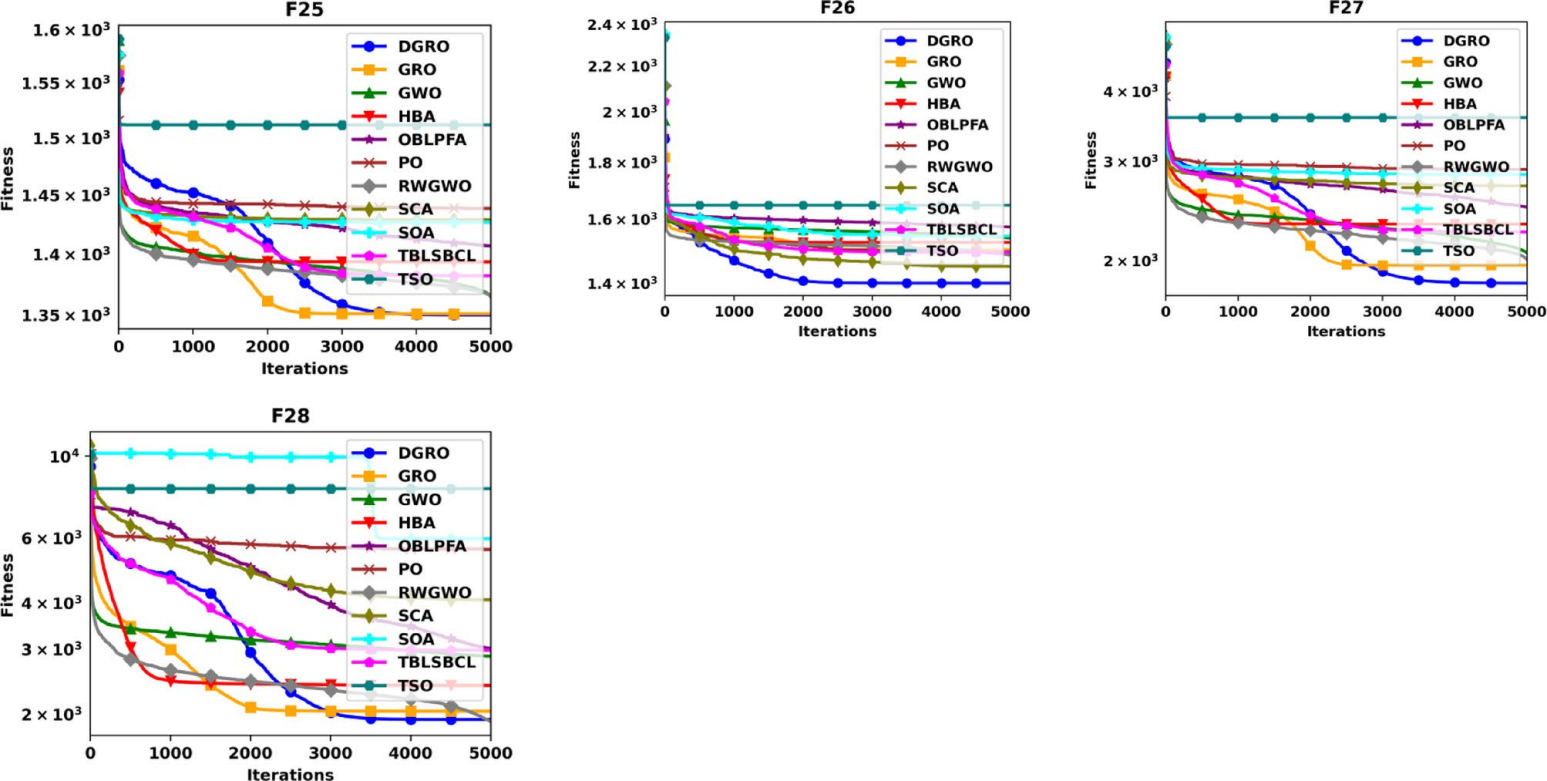
Convergence Chart Comparing DGRO with Other Optimizers on the CEC2013 Benchmark at 30 Dimensions.

**Fig. 3:**
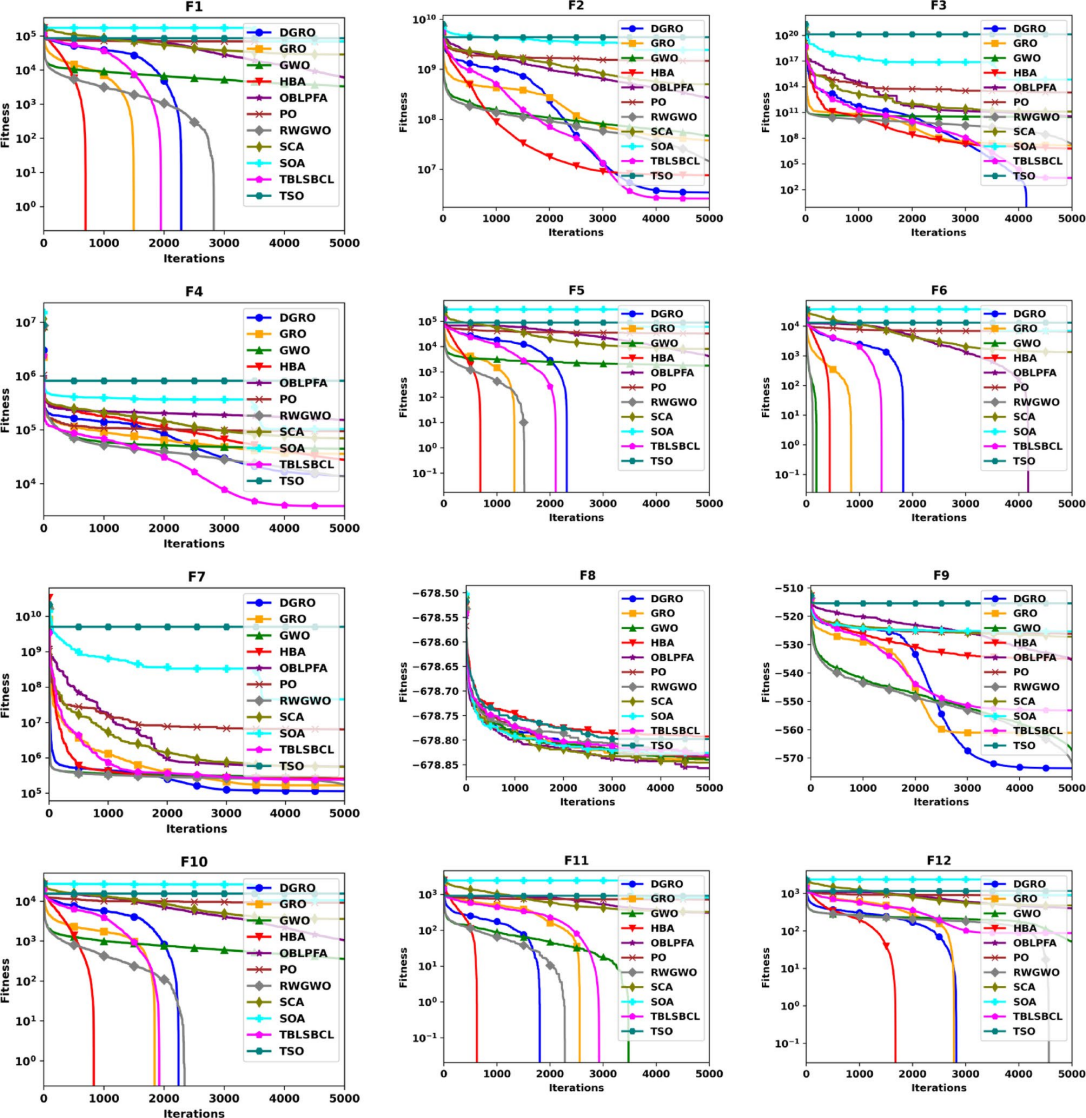

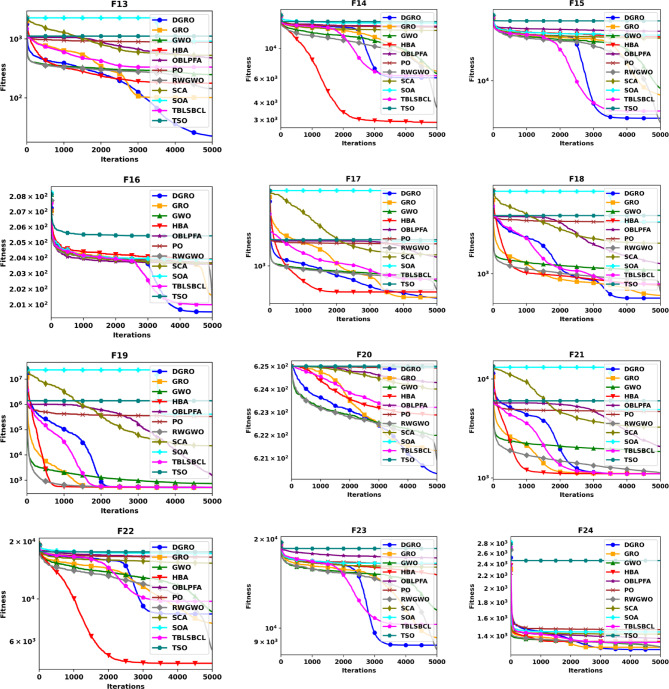

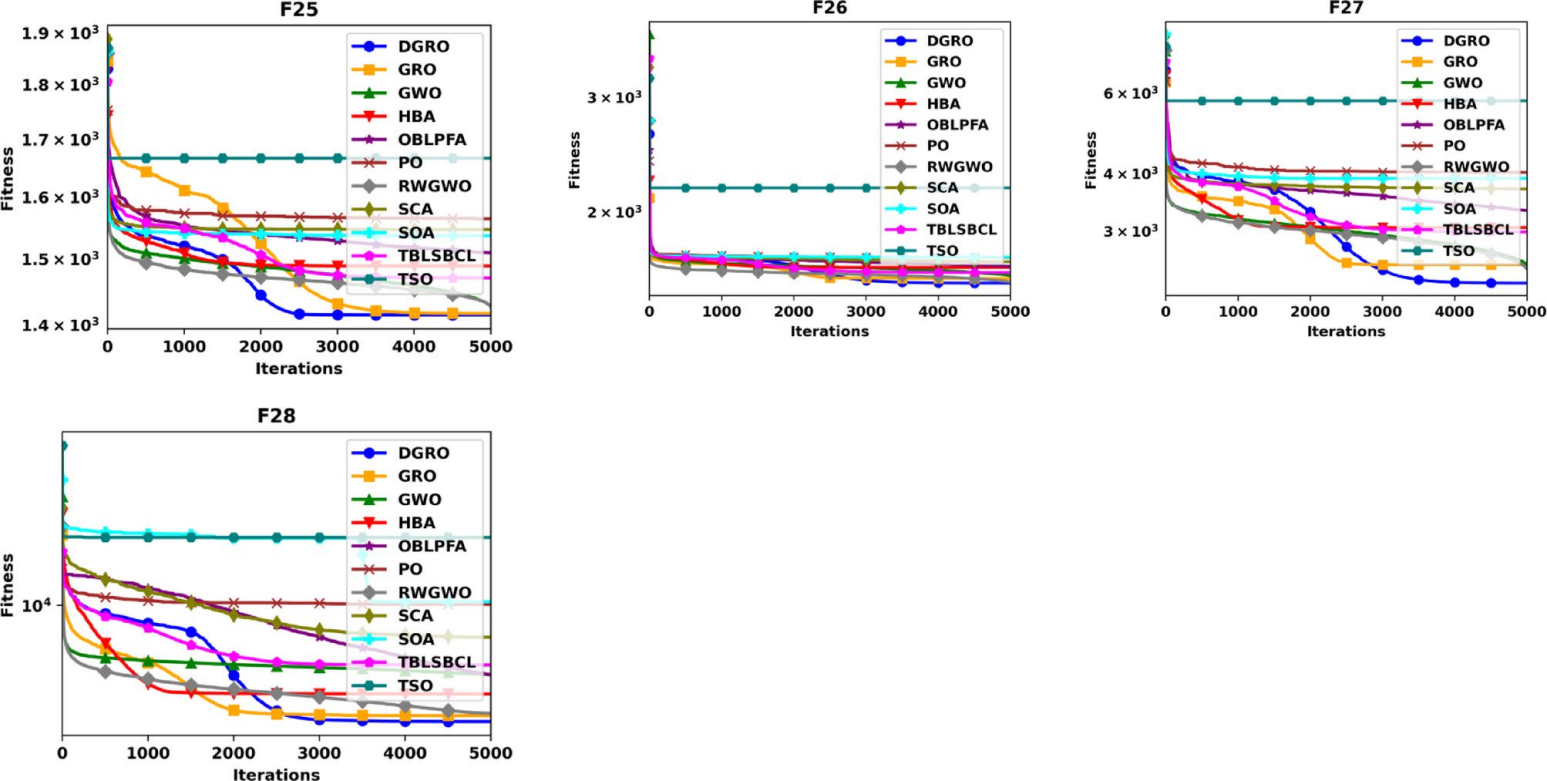
Convergence Chart Comparing DGRO with Other Optimizers on the CEC2013 Benchmark at 50 Dimensions.

To thoroughly evaluate the optimization efficacy of the proposed DGRO, experiments were conducted on benchmark functions in both 30 and 50 dimensions. The results are presented in Tables 2 and 3. Analyzing the findings in Table 2 indicates that DGRO outperforms GRO in most unimodal functions (F1–F5), with notable results in obtaining the most optimal solutions for F2 and F3 compared to other algorithms. Furthermore, DGRO achieves the global optimum for F1 and F5, matching the performance of advanced optimizers such as OBLPFA, RWGWO, TBLSBCL, and the traditional GRO. This performance demonstrate DGRO improved exploitative capability due to the introduction of SNM strategy. In the multimodal function set, DGRO demonstrates superior performance in F6, F7, F9, F10–F13, and F15–F20, while OBLPFA achieved outstanding performance for F8 and HBA outperforms in F14. These results align with the NFL theorem, which asserts that no single algorithm can achieve optimal solutions for all problems. The F6-F20 tests rigorously evaluate the exploration strategies employed by optimization algorithms. As evidenced by the results, the WAM strategy significantly enhances the exploration capabilities of DGRO, demonstrating its potential to achieve superior performance in complex optimization scenarios. For composite functions (F21–F28), DGRO achieves superior mean solutions in F21, and F24–F27, further emphasizing its robust performance. These results underscore the significant enhancements observed in the performance of DGRO in addressing complex functions effectively. Table 3 presents the results for 50 dimensions, which indicate similar performance as Table 2, reaffirming DGRO’s robustness and stability in both low and high-dimensional search spaces. Specifically, DGRO attains the globally optimal solutions for F1 and F5, comparable to improved optimizers such as RWGWO and TBLSBCL, as well as the traditional GRO. Additionally, DGRO achieves the most optimal solutions in F3 and F6. In multimodal functions, DGRO and TBLSBCL attained the most optimal solution for F10, while DGRO demonstrates superior performance in F7, F9, F11–F14, and F16–F20. For composite functions, DGRO achieved outstanding performance in F21 and F24–F28, indicating its adaptability and enhanced exploration capabilities. The improvement in DGRO’s performance is attributed to the integration of the SNM and WAM strategies, which enhance its ability to balance exploration and exploitation effectively. The convergence curves presented in Figs. 2 and 3 further illustrate DGRO’s superior convergence trajectory compared to other algorithms. These enhancements highlight DGRO’s improved search efficiency and its ability to avoid stagnation in local optima. Conclusively, DGRO demonstrates exceptional searchability and resilience against local optima across unimodal, multimodal, and composition test functions. The algorithm consistently achieves improved optimization performance, confirming its robustness and effectiveness as a competitive MA.

**Table 2:** Experimental results comparing DGRO with other optimizers on the CEC2013 benchmark at 30 dimensions.

		DGRO	GRO	GWO	HBA	OBLPFA	PO	RWGWO	SCA	SOA	TBLSBCL	TSO
F1	AVG	**-1.400E + 3**	**-1.400E + 3**	1.588E + 2	**-1.400E + 3**	8.684E + 2	4.020E + 4	**-1.400E + 3**	1.136E + 4	4.039E + 4	**-1.400E + 3**	6.221E + 4
	STD	9.003E-10	**1.340E-10**	8.468E + 2	4.298E-10	3.280E + 2	4.840E + 3	3.028E-2	2.176E + 3	6.391E + 3	8.895E-10	6.211E + 3
F2	AVG	**1.563E + 6**	1.187E + 7	2.781E + 7	3.457E + 6	7.668E + 7	4.519E + 8	5.550E + 6	1.729E + 8	9.093E + 8	1.681E + 6	2.483E + 9
	STD	7.537E + 5	4.296E + 6	1.668E + 7	2.199E + 6	2.807E + 7	1.288E + 8	2.587E + 6	5.620E + 7	3.758E + 8	**6.459E + 5**	7.698E + 8
F3	AVG	**-1.189E + 3**	-1.109E + 3	1.273E + 10	7.826E + 3	7.318E + 9	2.569E + 13	4.720E + 4	4.557E + 10	3.571E + 14	-6.509E + 2	1.726E + 21
	STD	1.821E + 1	1.395E + 2	6.687E + 9	**-2.450E + 4**	2.181E + 9	1.213E + 13	2.570E + 4	1.556E + 10	1.590E + 14	9.413E + 2	8.538E + 20
F4	AVG	3.605E + 3	1.667E + 4	2.893E + 4	1.067E + 4	5.733E + 4	6.180E + 4	4.889E + 3	3.659E + 4	6.741E + 4	**-1.633E + 2**	6.914E + 4
	STD	1.902E + 3	4.368E + 3	8.348E + 3	4.625E + 3	7.736E + 3	3.882E + 3	2.657E + 3	6.528E + 3	1.990E + 3	7.484E + 2	**7.392E + 1**
F5	AVG	**-1.000E + 3**	**-1.000E + 3**	7.245E + 2	**-1.000E + 3**	9.307E + 2	2.681E + 4	**-1.000E + 3**	5.187E + 3	6.006E + 4	**-1.000E + 3**	1.132E + 5
	STD	4.390E-5	3.689E-6	2.823E + 2	**2.787E-9**	4.896E + 2	6.720E + 3	1.354E-2	1.550E + 3	3.931E + 4	5.326E-5	4.396E + 4
F6	AVG	**-8.640E + 2**	-8.539E + 2	-7.544E + 2	-8.500E + 2	-6.866E + 2	4.888E + 3	-8.526E + 2	-1.744E + 2	6.182E + 3	-8.513E + 2	1.695E + 4
	STD	**1.603E + 1**	2.758E + 1	4.281E + 1	2.635E + 1	4.336E + 1	1.344E + 3	2.378E + 1	1.919E + 2	2.648E + 3	2.830E + 1	4.695E + 3
F7	AVG	**1.003E + 4**	2.656E + 4	6.171E + 4	7.899E + 4	1.350E + 5	2.318E + 6	3.654E + 4	1.541E + 5	8.522E + 6	7.472E + 4	9.137E + 9
	STD	**3.908E + 3**	1.336E + 4	2.271E + 4	1.871E + 4	3.203E + 4	2.153E + 6	1.610E + 4	2.851E + 4	6.141E + 6	1.662E + 4	5.661E + 9
F8	AVG	-6.790E + 2	-6.790E + 2	-6.790E + 2	-6.790E + 2	**-6.791E + 2**	-6.790E + 2	-6.790E + 2	-6.790E + 2	-6.790E + 2	-6.790E + 2	-6.790E + 2
	STD	5.175E-2	5.156E-2	4.073E-2	7.106E-2	4.813E-2	3.814E-2	7.022E-2	6.153E-2	6.087E-2	**3.677E-2**	5.166E-2
F9	AVG	**-5.893E + 2**	-5.844E + 2	-5.844E + 2	-5.684E + 2	-5.675E + 2	-5.603E + 2	-5.868E + 2	-5.608E + 2	-5.593E + 2	-5.784E + 2	-5.521E + 2
	STD	**9.088E-1**	5.249	3.066	2.983	2.695	1.355	2.910	1.419	5.185	2.988	1.679
F10	AVG	**-5.000E + 2**	-4.986E + 2	-1.652E + 2	-4.998E + 2	-3.084E + 1	4.729E + 3	-4.984E + 2	1.279E + 3	6.527E + 3	-4.998E + 2	1.181E + 4
	STD	**2.404E-2**	8.064E-1	1.595E + 2	1.777E-1	1.173E + 2	9.930E + 2	5.288E-1	3.539E + 2	1.832E + 3	8.080E-2	2.629E + 3
F11	AVG	**-3.623E + 2**	-3.336E + 2	-2.960E + 2	-3.402E + 2	-7.252E + 1	3.076E + 2	-3.506E + 2	-2.325E + 1	3.708E + 2	-2.465E + 2	6.760E + 2
	STD	**1.176E + 1**	1.505E + 1	3.194E + 1	2.339E + 1	3.254E + 1	5.573E + 1	1.327E + 1	4.012E + 1	1.062E + 2	3.156E + 1	6.744E + 1
F12	AVG	**-2.590E + 2**	-2.300E + 2	-1.266E + 2	-2.044E + 2	1.348E + 1	3.850E + 2	-2.085E + 2	8.845E + 1	4.110E + 2	-1.303E + 2	7.576E + 2
	STD	9.663	1.728E + 1	6.341E + 1	2.891E + 1	**7.494**	6.703E + 1	1.964E + 1	3.201E + 1	8.238E + 1	4.777E + 1	9.132E + 1
F13	AVG	**-1.035E + 2**	-5.067E + 1	-1.337	-3.351E + 1	1.029E + 2	4.428E + 2	-3.116E + 1	1.749E + 2	5.115E + 2	6.598E + 1	7.926E + 2
	STD	**2.634E + 1**	2.974E + 1	4.668E + 1	3.170E + 1	2.882E + 1	5.996E + 1	3.947E + 1	3.128E + 1	8.518E + 1	6.153E + 1	8.662E + 1
F14	AVG	3.282E + 3	3.004E + 3	4.022E + 3	**1.343E + 3**	7.963E + 3	7.445E + 3	1.688E + 3	7.151E + 3	8.469E + 3	3.497E + 3	9.388E + 3
	STD	6.299E + 2	6.157E + 2	2.028E + 3	3.648E + 2	4.253E + 2	3.529E + 2	4.743E + 2	4.387E + 2	**2.962E + 2**	5.387E + 2	3.773E + 2
F15	AVG	**3.500E + 3**	3.625E + 3	5.281E + 3	7.353E + 3	7.884E + 3	7.711E + 3	3.513E + 3	7.619E + 3	8.017E + 3	3.698E + 3	9.876E + 3
	STD	5.963E + 2	4.931E + 2	2.065E + 3	7.955E + 2	3.178E + 2	2.561E + 2	6.763E + 2	**2.537E + 2**	3.346E + 2	5.769E + 2	4.656E + 2
F16	AVG	**2.003E + 2**	2.008E + 2	2.027E + 2	2.028E + 2	2.026E + 2	2.026E + 2	2.007E + 2	2.026E + 2	2.028E + 2	2.006E + 2	2.045E + 2
	STD	1.966E-1	2.468E-1	2.881E-1	3.319E-1	3.132E-1	3.426E-1	3.036E-1	3.591E-1	3.538E-1	2.681E-1	**1.279E-1**
F17	AVG	**3.715E + 2**	3.939E + 2	5.048E + 2	4.022E + 2	7.352E + 2	1.094E + 3	4.071E + 2	8.159E + 2	1.273E + 3	4.757E + 2	1.311E + 3
	STD	**1.235E + 1**	1.797E + 1	4.420E + 1	1.605E + 1	4.378E + 1	6.079E + 1	1.789E + 1	5.060E + 1	6.242E + 1	3.322E + 1	2.637E + 1
F18	AVG	**4.632E + 2**	4.673E + 2	6.557E + 2	5.209E + 2	8.101E + 2	2.352E + 3	4.934E + 2	1.201E + 3	2.455E + 3	5.451E + 2	3.236E + 3
	STD	**1.476E + 1**	1.554E + 1	4.720E + 1	4.649E + 1	4.046E + 1	1.833E + 2	2.309E + 1	1.097E + 2	3.192E + 2	5.430E + 1	1.836E + 2
F19	AVG	**5.052E + 2**	5.053E + 2	5.391E + 2	5.082E + 2	5.750E + 2	2.690E + 5	5.061E + 2	6.114E + 3	6.001E + 5	5.088E + 2	5.948E + 6
	STD	**1.156**	1.890	3.670E + 1	3.800	3.770E + 1	1.120E + 5	1.872	3.910E + 3	5.810E + 5	2.439	4.443E + 6
F20	AVG	**6.108E + 2**	6.115E + 2	6.131E + 2	6.134E + 2	6.139E + 2	6.150E + 2	6.120E + 2	6.140E + 2	6.149E + 2	6.138E + 2	6.150E + 2
	STD	5.476E-1	5.680E-1	1.061	9.790E-1	5.281E-1	5.261E-2	1.386	3.058E-1	1.524E-1	1.321	**2.728E-13**
F21	AVG	**1.146E + 3**	1.245E + 3	1.966E + 3	1.703E + 3	1.997E + 3	3.234E + 3	1.268E + 3	2.454E + 3	3.486E + 3	1.688E + 3	4.196E + 3
	STD	1.909E + 2	3.030E + 2	1.514E + 2	3.110E + 2	**8.509E + 1**	1.642E + 2	3.211E + 2	1.078E + 2	3.004E + 2	3.080E + 2	2.294E + 2
F22	AVG	4.883E + 3	3.828E + 3	4.179E + 3	**2.282E + 3**	9.338E + 3	9.307E + 3	2.508E + 3	8.497E + 3	1.018E + 4	5.494E + 3	1.025E + 4
	STD	8.369E + 2	5.898E + 2	1.222E + 3	3.458E + 2	6.166E + 2	2.599E + 2	4.050E + 2	3.973E + 2	3.355E + 2	7.509E + 2	**7.758E + 1**
F23	AVG	4.543E + 3	4.429E + 3	5.574E + 3	7.711E + 3	9.594E + 3	9.014E + 3	**4.354E + 3**	8.779E + 3	9.203E + 3	5.179E + 3	1.114E + 4
	STD	6.052E + 2	4.745E + 2	1.864E + 3	1.289E + 3	4.577E + 2	**3.373E + 2**	6.616E + 2	3.599E + 2	4.593E + 2	8.513E + 2	5.070E + 2
F24	AVG	**1.218E + 3**	1.228E + 3	1.240E + 3	1.265E + 3	1.289E + 3	1.331E + 3	1.239E + 3	1.310E + 3	1.324E + 3	1.257E + 3	1.559E + 3
	STD	5.923	1.378E + 1	8.033	1.055E + 1	8.815	5.797	7.484	**4.984**	6.367	1.138E + 1	1.904E + 2
F25	AVG	**1.350E + 3**	1.351E + 3	1.365E + 3	1.393E + 3	1.407E + 3	1.438E + 3	1.364E + 3	1.429E + 3	1.427E + 3	1.382E + 3	1.512E + 3
	STD	1.040E + 1	1.377E + 1	7.882	1.101E + 1	1.013E + 1	7.346	1.157E + 1	4.365	**3.932**	8.800	3.243E + 1
F26	AVG	**1.400E + 3**	1.501E + 3	1.537E + 3	1.524E + 3	1.574E + 3	1.494E + 3	1.483E + 3	1.450E + 3	1.544E + 3	1.492E + 3	1.646E + 3
	STD	**3.896E-2**	6.228E + 1	3.078E + 1	7.622E + 1	4.616E + 1	2.600E + 1	6.899E + 1	6.824E + 1	5.250E + 1	7.737E + 1	9.476
F27	AVG	**1.822E + 3**	1.960E + 3	2.061E + 3	2.321E + 3	2.490E + 3	2.905E + 3	2.005E + 3	2.715E + 3	2.844E + 3	2.247E + 3	3.590E + 3
	STD	8.167E + 1	1.446E + 2	9.509E + 1	1.035E + 2	7.734E + 1	**6.863E + 1**	9.670E + 1	7.166E + 1	8.440E + 1	9.069E + 1	4.564E + 2
F28	AVG	1.933E + 3	2.035E + 3	2.865E + 3	2.391E + 3	3.007E + 3	5.575E + 3	**1.908E + 3**	4.080E + 3	5.966E + 3	2.978E + 3	8.149E + 3
	STD	3.352E + 2	3.659E + 2	3.179E + 2	6.185E + 2	2.213E + 2	3.237E + 2	3.105E + 2	**2.094E + 2**	5.496E + 2	4.150E + 2	1.281E + 3

**Table 3:** Experimental results comparing DGRO with other optimizers on the CEC2013 benchmark at 50 dimensions.

		DGRO	GRO	GWO	HBA	OBLPFA	PO	RWGWO	SCA	SOA	TBLSBCL	TSO
F1	AVG	**-1.400E + 3**	**-1.400E + 3**	3.369E + 3	**-1.400E + 3**	6.060E + 3	6.912E + 4	**-1.400E + 3**	2.879E + 4	6.990E + 4	**-1.400E + 3**	8.645E + 4
	STD	**0**	6.129E-3	2.258E + 3	8.032E-4	1.166E + 3	5.035E + 3	2.430E-1	3.824E + 3	6.295E + 3	2.695E-9	3.098E + 3
F2	AVG	3.428E + 6	3.768E + 7	4.655E + 7	7.598E + 6	2.663E + 8	1.472E + 9	1.450E + 7	5.034E + 8	2.439E + 9	**2.607E + 6**	4.383E + 9
	STD	1.263E + 6	1.715E + 7	1.800E + 7	3.544E + 6	5.004E + 7	2.823E + 8	5.874E + 6	1.469E + 8	9.912E + 8	**9.337E + 5**	1.138E + 9
F3	AVG	**-7.533E + 2**	1.468E + 7	3.031E + 10	6.559E + 6	3.758E + 10	2.156E + 13	2.204E + 7	1.291E + 11	7.321E + 14	2.433E + 3	1.350E + 20
	STD	1.333E + 3	1.190E + 6	8.587E + 9	3.745E + 4	7.657E + 9	1.587E + 13	1.450E + 7	6.061E + 10	1.753E + 13	**3.627E + 2**	1.100E + 20
F4	AVG	1.378E + 4	3.586E + 4	4.460E + 4	2.775E + 4	1.528E + 5	9.468E + 4	1.370E + 4	6.926E + 4	1.035E + 5	**3.833E + 3**	8.163E + 5
	STD	4.635E + 3	6.701E + 3	9.371E + 3	6.894E + 3	3.833E + 4	6.940E + 3	5.294E + 3	7.643E + 3	1.708E + 4	**1.510E + 3**	7.828E + 5
F5	AVG	**-1.000E + 3**	-9.999E + 2	1.786E + 3	**-1.000E + 3**	4.214E + 3	3.266E + 4	-9.994E + 2	8.176E + 3	6.139E + 4	**-1.000E + 3**	8.958E + 4
	STD	1.130E-4	1.262E-1	1.725E + 3	7.217E-3	1.019E + 3	7.221E + 3	1.908	2.086E + 3	2.174E + 4	**8.706E-5**	1.570E + 4
F6	AVG	**-8.522E + 2**	-8.521E + 2	-5.694E + 2	-8.256E + 2	-3.981E + 2	6.624E + 3	-8.497E + 2	1.339E + 3	7.044E + 3	-8.352E + 2	1.313E + 4
	STD	**9.324E-1**	1.005E + 1	9.266E + 1	2.966E + 1	1.105E + 2	1.114E + 3	1.251E + 1	4.135E + 2	1.338E + 3	2.558E + 1	1.858E + 3
F7	AVG	**1.149E + 5**	1.681E + 5	2.434E + 5	2.629E + 5	5.708E + 5	6.368E + 6	1.766E + 5	5.627E + 5	4.538E + 7	2.437E + 5	5.057E + 9
	STD	**2.482E + 4**	3.092E + 4	6.117E + 4	5.566E + 4	6.860E + 4	6.260E + 6	5.280E + 4	6.590E + 4	3.375E + 6	2.720E + 4	2.233E + 8
F8	AVG	-6.788E + 2	-6.788E + 2	-6.788E + 2	-6.788E + 2	**-6.789E + 2**	-6.788E + 2	-6.788E + 2	-6.788E + 2	-6.788E + 2	-6.788E + 2	-6.788E + 2
	STD	4.179E-2	3.774E-2	3.896E-2	2.929E-2	4.576E-2	**2.784E-2**	3.550E-2	3.050E-2	3.216E-2	2.994E-2	3.464E-2
F9	AVG	**-5.736E + 2**	-5.611E + 2	-5.673E + 2	-5.350E + 2	-5.354E + 2	-5.262E + 2	-5.716E + 2	-5.272E + 2	-5.253E + 2	-5.532E + 2	-5.154E + 2
	STD	6.600	8.790	4.529	4.082	4.200	**1.553**	4.938	2.111	1.652	4.751	1.891
F10	AVG	**-4.999E + 2**	-4.705E + 2	3.587E + 2	-4.954E + 2	1.048E + 3	9.220E + 3	-4.885E + 2	3.615E + 3	1.064E + 4	**-4.999E + 2**	1.555E + 4
	STD	**3.417E-2**	1.218E + 1	2.656E + 2	2.138	2.759E + 2	1.578E + 3	5.368	5.185E + 2	1.842E + 3	3.999E-2	1.758E + 3
F11	AVG	**-3.056E + 2**	-2.640E + 2	-1.278E + 2	-2.425E + 2	3.060E + 2	7.201E + 2	-2.785E + 2	3.271E + 2	8.073E + 2	-2.374E + 1	9.161E + 2
	STD	**2.015E + 1**	3.480E + 1	5.755E + 1	3.415E + 1	7.531E + 1	5.311E + 1	2.623E + 1	4.989E + 1	9.096E + 1	4.445E + 1	3.097E + 1
F12	AVG	**-1.752E + 2**	-1.310E + 2	5.204E + 1	-8.713E + 1	4.037E + 2	8.961E + 2	-1.146E + 2	4.806E + 2	9.115E + 2	8.800E + 1	1.174E + 3
	STD	**2.675E + 1**	3.034E + 1	4.917E + 1	5.688E + 1	7.791E + 1	6.421E + 1	3.770E + 1	6.660E + 1	8.035E + 1	7.845E + 1	8.465E + 1
F13	AVG	**2.252E + 1**	1.017E + 2	2.479E + 2	1.772E + 2	4.771E + 2	8.756E + 2	1.442E + 2	5.250E + 2	9.163E + 2	3.326E + 2	1.112E + 3
	STD	**5.886**	4.001E + 1	7.574E + 1	6.306E + 1	7.523E + 1	8.084E + 1	5.514E + 1	4.246E + 1	8.252E + 1	7.444E + 1	6.668E + 1
F14	AVG	**2.870E + 3**	6.789E + 3	6.534E + 3	6.084E + 3	1.452E + 4	1.427E + 4	3.682E + 3	1.352E + 4	1.531E + 4	6.307E + 3	1.570E + 4
	STD	8.909E + 2	9.726E + 2	2.572E + 3	6.310E + 2	5.252E + 2	3.365E + 2	5.519E + 2	4.813E + 2	**2.965E + 2**	1.000E + 3	3.538E + 2
F15	AVG	7.127E + 3	8.787E + 3	9.348E + 3	1.489E + 4	1.564E + 4	1.471E + 4	**6.862E + 3**	1.469E + 4	1.511E + 4	7.608E + 3	1.718E + 4
	STD	7.362E + 2	1.051E + 3	3.787E + 3	4.335E + 2	3.885E + 2	3.387E + 2	1.065E + 3	**3.317E + 2**	4.670E + 2	8.808E + 2	3.865E + 2
F16	AVG	**2.005E + 2**	2.016E + 2	2.037E + 2	2.039E + 2	2.036E + 2	2.037E + 2	2.010E + 2	2.036E + 2	2.039E + 2	2.010E + 2	2.054E + 2
	STD	2.803E-1	3.900E-1	3.556E-1	4.063E-1	4.201E-1	3.403E-1	3.754E-1	2.637E-1	3.139E-1	3.967E-1	**8.781E-2**
F17	AVG	**4.676E + 2**	4.766E + 2	6.972E + 2	5.400E + 2	1.232E + 3	1.685E + 3	5.633E + 2	1.301E + 3	1.783E + 3	7.326E + 2	1.857E + 3
	STD	2.668E + 1	3.204E + 1	8.545E + 1	4.453E + 1	9.232E + 1	6.618E + 1	4.574E + 1	7.966E + 1	6.518E + 1	9.940E + 1	**1.516E + 1**
F18	AVG	**5.388E + 2**	5.760E + 2	1.096E + 3	7.498E + 2	1.293E + 3	3.698E + 3	6.424E + 2	2.173E + 3	3.735E + 3	7.739E + 2	4.379E + 3
	STD	**2.476E + 1**	3.044E + 1	1.147E + 2	9.970E + 1	6.442E + 1	1.707E + 2	3.880E + 1	1.776E + 2	2.860E + 2	7.602E + 1	1.342E + 2
F19	AVG	**5.103E + 2**	5.132E + 2	7.331E + 2	5.267E + 2	1.549E + 3	3.337E + 5	5.128E + 2	2.322E + 4	4.093E + 5	5.225E + 2	1.395E + 6
	STD	2.512	**2**	2.686E + 2	7.426	7.743E + 2	9.546E + 4	3.058	1.030E + 4	2.129E + 5	6.298	4.123E + 5
F20	AVG	**6.203E + 2**	6.213E + 2	6.220E + 2	6.229E + 2	6.243E + 2	6.249E + 2	6.209E + 2	6.240E + 2	6.250E + 2	6.232E + 2	6.250E + 2
	STD	6.061E-1	7.657E-1	5.619E-1	1.197	5.575E-1	1.095E-1	8.403E-1	3.247E-1	9.216E-2	1.086	**3.081E-10**
F21	AVG	**1.100E + 3**	1.105E + 3	1.849E + 3	1.101E + 3	2.067E + 3	4.686E + 3	1.127E + 3	3.259E + 3	4.907E + 3	**1.100E + 3**	6.017E + 3
	STD	**1.793E-4**	4.072	2.315E + 2	2.400	1.086E + 2	1.686E + 2	3.299	1.765E + 2	3.299E + 2	2.033E-4	6.411E + 2
F22	AVG	8.340E + 3	7.443E + 3	8.574E + 3	**4.599E + 3**	1.655E + 4	1.656E + 4	5.411E + 3	1.540E + 4	1.729E + 4	9.717E + 3	1.766E + 4
	STD	1.049E + 3	9.680E + 2	2.383E + 3	7.206E + 2	7.465E + 2	3.583E + 2	7.403E + 2	5.021E + 2	5.061E + 2	1.349E + 3	**4.313E + 1**
F23	AVG	8.759E + 3	9.267E + 3	1.151E + 4	1.515E + 4	1.727E + 4	1.645E + 4	**8.471E + 3**	1.608E + 4	1.658E + 4	1.030E + 4	1.858E + 4
	STD	9.587E + 2	1.008E + 3	3.465E + 3	1.091E + 3	4.921E + 2	3.275E + 2	1.392E + 3	4.351E + 2	4.229E + 2	1.201E + 3	**3.161E + 2**
F24	AVG	**1.264E + 3**	1.286E + 3	1.289E + 3	1.343E + 3	1.373E + 3	1.468E + 3	1.286E + 3	1.419E + 3	1.442E + 3	1.334E + 3	2.461E + 3
	STD	1.063E + 1	2.238E + 1	1.002E + 1	1.369E + 1	8.806	2.018E + 1	1.688E + 1	**6.555**	1.347E + 1	1.405E + 1	6.601E + 2
F25	AVG	**1.414E + 3**	1.417E + 3	1.427E + 3	1.489E + 3	1.509E + 3	1.564E + 3	1.426E + 3	1.546E + 3	1.537E + 3	1.470E + 3	1.666E + 3
	STD	1.887E + 1	1.298E + 1	1.420E + 1	1.353E + 1	1.762E + 1	1.632E + 1	1.491E + 1	**7.690**	8.670	1.504E + 1	5.224E + 1
F26	AVG	**1.556E + 3**	1.581E + 3	1.597E + 3	1.644E + 3	1.651E + 3	1.703E + 3	1.560E + 3	1.678E + 3	1.705E + 3	1.615E + 3	2.177E + 3
	STD	3.162E + 1	5.500E + 1	5.624E + 1	1.485E + 1	4.472E + 1	9.561	4.477E + 1	5.117E + 1	**7.503**	1.339E + 1	8.362E + 2
F27	AVG	**2.306E + 3**	2.529E + 3	2.530E + 3	3.050E + 3	3.323E + 3	4.024E + 3	2.474E + 3	3.701E + 3	3.905E + 3	2.983E + 3	5.762E + 3
	STD	1.293E + 2	2.120E + 2	1.013E + 2	1.179E + 2	1.207E + 2	1.248E + 2	1.175E + 2	**5.603E + 1**	7.514E + 1	1.299E + 2	1.098E + 3
F28	AVG	**2.561E + 3**	2.746E + 3	4.460E + 3	3.537E + 3	4.430E + 3	1.012E + 4	2.809E + 3	6.894E + 3	1.042E + 4	4.975E + 3	2.217E + 4
	STD	**2.809E + 2**	4.603E + 2	7.182E + 2	1.158E + 3	3.160E + 2	5.270E + 2	4.929E + 2	3.740E + 2	1.255E + 3	7.423E + 2	7.666E + 3

### Experimental results of CEC2020

**Fig. 4:**
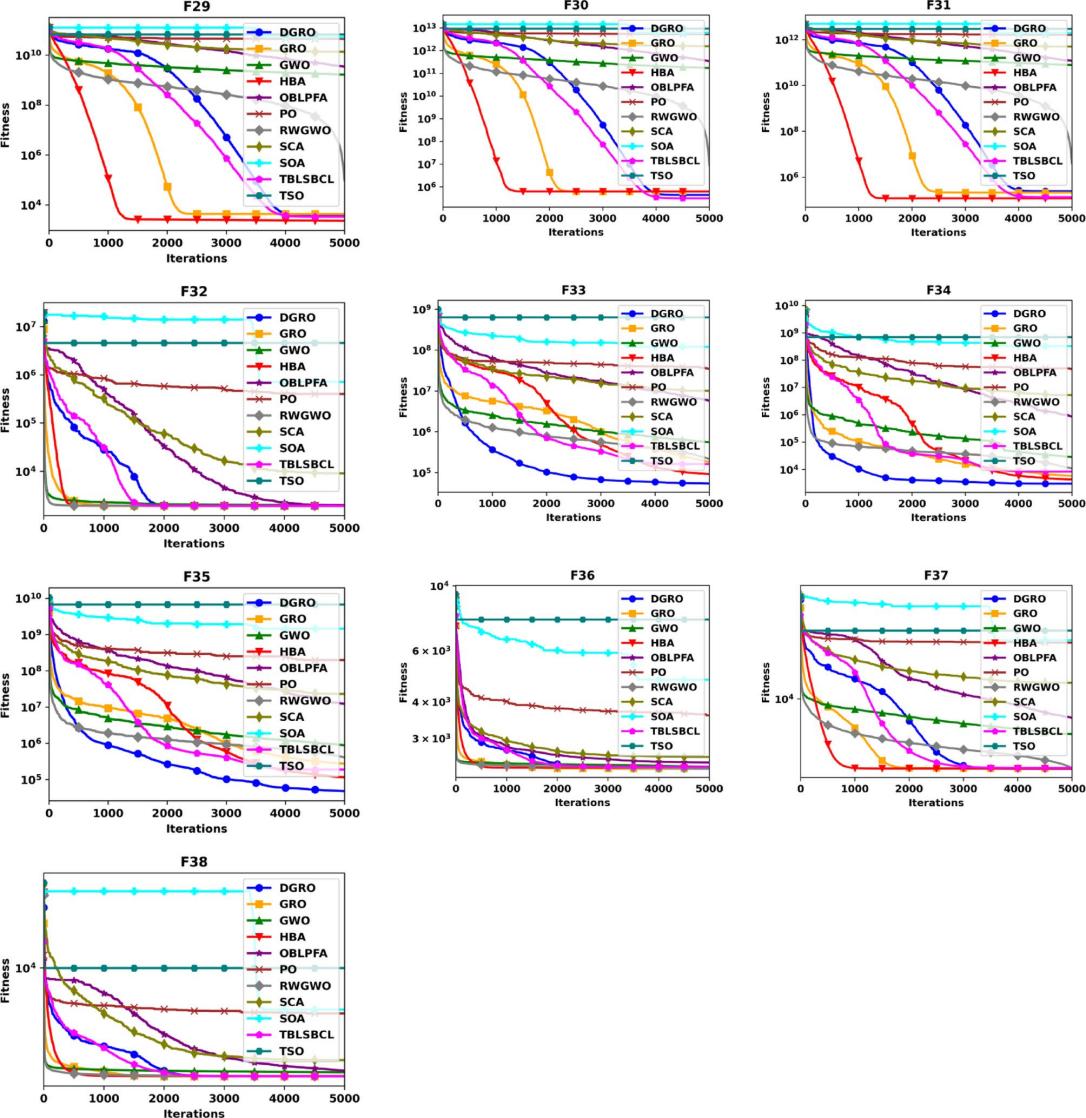
Convergence Chart Comparing DGRO with Other Optimizers on the CEC2020 Benchmark at 30 Dimensions.

**Fig. 5:**
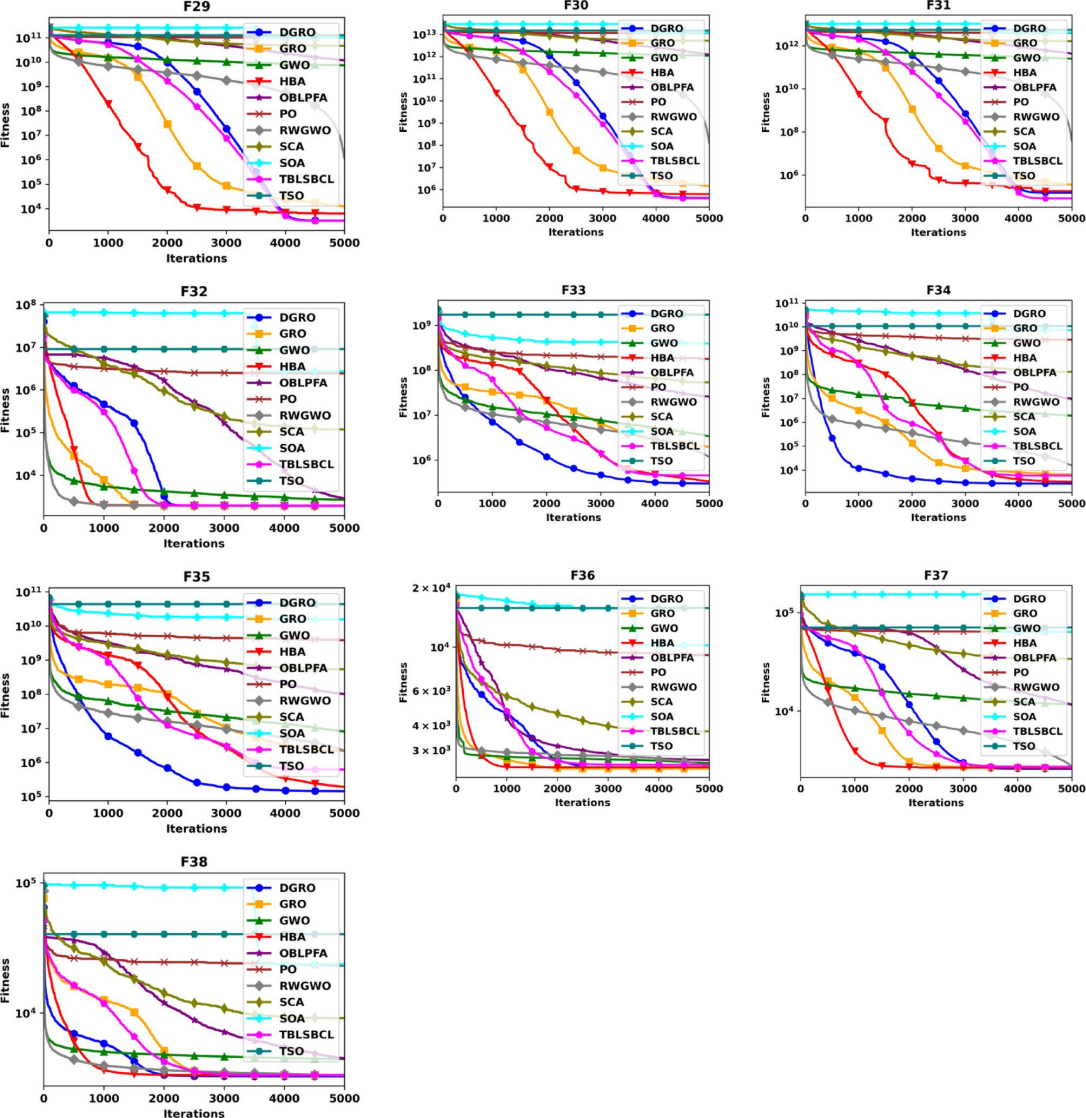
Convergence Chart Comparing DGRO with Other Optimizers on the CEC2020 Benchmark at 50 Dimensions.

This section provides a comparative analysis of DGRO against various MAs on the CEC2020 benchmark set, evaluated for both 30 and 50 dimensions. The statistical results are presented in Tables 4 and 5, respectively. As shown in Table 4, DGRO demonstrates its effectiveness in optimizing the benchmark functions, outperforming other algorithms, while maintain similar performance to GRO on F32 from the multimodal functions. For the hybrid functions (F33–F35), DGRO exhibits superior performance compared to OBLPFA, RWGWO, and TBLSBCL. Similarly, DGRO achieves enhanced performance in F36 and F37, which are composite functions, although the traditional GRO achieves a marginally more optimal solution on F38. The results for the more complex 50 dimension CEC2020, presented in Table 5, demonstrate DGRO maintains consistent performance. It achieves the most optimal solution on F32, performing both the traditional GRO and improved algorithms, including RWGWO and TBLSBCL. Additionally, DGRO demonstrates superior performance on the hybrid functions (F33–F35) and composite functions (F37 and F38), further affirming its robustness and adaptability to complex optimization scenarios. The analysis of functions F29–F31 validates the NFL theorem, which states that no single algorithm can provide the most optimal solution across all problems. While DGRO achieved superior performance in the majority of these functions, algorithms, such as HBA and TBLSBCL, achieved optimal results on a few functions, highlighting the competitive nature of DGRO’s performance. The overall results strongly support the effectiveness and optimization capabilities of the proposed DGRO approach. Its robust performance in handling complex function sets is further corroborated by the convergence characteristics depicted in Figs. 4 and 5, which showcase the convergence patterns of DGRO and the compared algorithms on the CEC2020 benchmark for 30 and 50 dimensions. A detailed examination of the plots underscores DGRO’s remarkable convergence capabilities, which is attributed to the strategic integration of the SNM and the WAM. These enhancements significantly improved DGRO’s ability to balance exploration and exploitation, expediting convergence and ensuring the identification of optimal solutions.

**Table 4:** Experimental results comparing DGRO with other optimizers on the CEC2020 benchmark at 30 dimensions.

		DGRO	GRO	GWO	HBA	OBLPFA	PO	RWGWO	SCA	SOA	TBLSBCL	TSO
F29	AVG	3.668E + 3	4.293E + 3	1.677E + 9	**2.309E + 3**	3.479E + 9	4.573E + 10	9.918E + 4	1.370E + 10	5.244E + 10	3.345E + 3	6.761E + 10
	STD	2.459E + 3	4.008E + 3	1.406E + 9	**1.756E + 3**	7.230E + 8	5.746E + 09	4.986E + 4	2.132E + 09	9.781E + 9	3.000E + 3	1.048E + 10
F30	AVG	4.377E + 5	6.279E + 5	1.714E + 11	6.338E + 5	3.432E + 11	5.286E + 12	8.697E + 6	1.564E + 12	6.097E + 12	**3.125E + 5**	9.276E + 12
	STD	3.486E + 5	5.710E + 5	1.428E + 11	4.761E + 5	6.564E + 10	8.553E + 11	7.418E + 6	1.950E + 11	1.231E + 12	**3.102E + 5**	1.308E + 12
F31	AVG	2.440E + 5	2.145E + 5	7.822E + 10	**1.187E + 5**	1.192E + 11	1.634E + 12	4.050E + 6	4.991E + 11	1.907E + 12	1.358E + 5	2.929E + 12
	STD	2.044E + 5	1.190E + 5	7.195E + 10	1.132E + 5	1.561E + 10	2.376E + 11	3.148E + 6	9.725E + 10	2.891E + 11	**4.354E + 4**	4.333E + 11
F32	AVG	**1.905E + 3**	**1.905E + 3**	1.973E + 3	1.910E + 3	1.964E + 3	4.021E + 5	1.906E + 3	9.070E + 3	7.148E + 5	1.909E + 3	4.581E + 6
	STD	1.468	1.311	1.324E + 2	3.455	4.156E + 1	1.919E + 5	**1.274**	6.169E + 3	6.926E + 5	2.882	2.126E + 6
F33	AVG	**5.406E + 4**	1.835E + 5	5.519E + 5	9.194E + 4	5.775E + 6	3.513E + 7	2.169E + 5	1.011E + 7	1.201E + 8	1.619E + 5	6.327E + 8
	STD	**2.645E + 4**	6.387E + 4	5.062E + 5	3.031E + 4	3.156E + 6	1.038E + 7	8.616E + 4	3.972E + 6	6.529E + 7	7.920E + 4	3.102E + 8
F34	AVG	**3.032E + 3**	5.806E + 3	2.875E + 4	4.331E + 3	8.711E + 5	4.840E + 7	1.099E + 4	5.324E + 6	3.301E + 8	8.394E + 3	6.986E + 8
	STD	**2.451E + 3**	3.988E + 3	1.132E + 4	3.909E + 3	5.335E + 5	3.834E + 7	7.488E + 3	2.273E + 6	2.972E + 8	4.409E + 3	3.326E + 8
F35	AVG	**4.804E + 4**	2.749E + 5	8.894E + 5	1.160E + 5	1.237E + 7	1.975E + 8	4.131E + 5	2.299E + 7	1.458E + 9	1.900E + 5	6.760E + 9
	STD	**4.378E + 4**	1.891E + 5	6.573E + 5	6.845E + 4	5.782E + 6	9.725E + 7	3.006E + 5	9.708E + 6	9.656E + 8	7.078E + 4	5.508E + 9
F36	AVG	**2.359E + 3**	2.361E + 3	2.398E + 3	2.377E + 3	2.478E + 3	3.597E + 3	**2.359E + 3**	2.589E + 3	4.759E + 3	2.395E + 3	7.665E + 3
	STD	2.375E + 1	1.540E + 1	1.375E + 1	**6.882**	1.810E + 1	2.745E + 2	2.010E + 1	3.614E + 1	1.233E + 3	2.560E + 1	1.360E + 3
F37	AVG	**2.600E + 3**	2.619E + 3	5.079E + 3	2.613E + 3	6.939E + 3	2.958E + 4	2.636E + 3	1.363E + 4	3.100E + 4	2.651E + 3	3.730E + 4
	STD	**5.670E-4**	4.964E + 1	1.710E + 3	5.017E + 1	3.899E + 2	3.726E + 3	5.552E + 1	1.044E + 3	4.924E + 3	1.156E + 2	1.453E + 3
F38	AVG	2.922E + 3	**2.921E + 3**	3.058E + 3	2.929E + 3	3.119E + 3	5.968E + 3	**2.921E + 3**	3.512E + 3	6.229E + 3	2.922E + 3	9.978E + 3
	STD	7.455E-1	**1.733E-1**	6.825E + 1	9.232	7.076E + 1	6.333E + 2	2.554E-1	1.596E + 2	9.713E + 2	3.853	9.924E + 2

**Table 5:** Experimental results comparing DGRO with other optimizers on the CEC2020 benchmark at 50 dimensions.

		DGRO	GRO	GWO	HBA	OBLPFA	PO	RWGWO	SCA	SOA	TBLSBCL	TSO
F29	AVG	3.273E + 3	1.233E + 4	7.490E + 9	6.324E + 3	1.201E + 10	1.022E + 11	1.149E + 6	4.750E + 10	1.043E + 11	**3.196E + 3**	1.276E + 11
	STD	**2.661E + 3**	1.072E + 4	3.711E + 9	6.258E + 3	1.768E + 9	7.702E + 9	6.109E + 5	6.242E + 9	9.044E + 9	2.729E + 3	5.938E + 9
F30	AVG	4.330E + 5	1.461E + 6	1.094E + 12	6.458E + 5	1.258E + 12	1.158E + 13	1.220E + 8	5.239E + 12	1.214E + 13	**4.118E + 5**	1.522E + 13
	STD	3.744E + 5	1.169E + 6	4.863E + 11	4.589E + 5	1.929E + 11	9.661E + 11	8.191E + 7	5.142E + 11	1.315E + 12	**3.402E + 5**	1.078E + 12
F31	AVG	1.530E + 5	3.611E + 5	2.496E + 11	1.795E + 5	4.252E + 11	3.953E + 12	3.777E + 7	1.627E + 12	4.107E + 12	**8.300E + 4**	5.431E + 12
	STD	1.454E + 5	2.615E + 5	1.375E + 11	1.538E + 5	4.953E + 10	3.143E + 11	2.087E + 7	2.181E + 11	6.168E + 11	**5.748E + 4**	4.122E + 11
F32	AVG	**1.910E + 3**	1.914E + 3	2.692E + 3	1.938E + 3	2.873E + 3	2.460E + 6	1.914E + 3	1.191E + 5	2.741E + 6	1.929E + 3	9.160E + 6
	STD	**2.237**	2.870	1.272E + 3	1.380E + 1	6.763E + 2	5.374E + 5	2.628	6.676E + 4	1.321E + 6	6.946	2.341E + 6
F33	AVG	**3.019E + 5**	1.975E + 6	3.393E + 6	3.355E + 5	2.557E + 7	1.780E + 8	1.192E + 6	5.388E + 7	3.971E + 8	4.596E + 5	1.724E + 9
	STD	2.586E + 5	1.062E + 6	1.823E + 6	1.637E + 5	8.922E + 6	5.862E + 7	4.746E + 5	1.829E + 7	1.347E + 8	**1.591E + 5**	6.900E + 8
F34	AVG	**2.705E + 3**	6.588E + 3	1.902E + 6	3.241E + 3	9.186E + 6	2.796E + 9	1.590E + 4	1.296E + 8	7.282E + 9	5.848E + 3	1.082E + 10
	STD	1.568E + 3	3.842E + 3	5.204E + 5	**7.339E + 2**	3.596E + 6	1.351E + 9	6.400E + 3	6.652E + 7	6.707E + 9	1.567E + 3	3.086E + 9
F35	AVG	**1.449E + 5**	2.189E + 6	8.195E + 6	1.917E + 5	1.021E + 8	3.880E + 9	2.239E + 6	5.463E + 8	1.579E + 10	6.263E + 5	4.416E + 10
	STD	**7.822E + 4**	1.802E + 6	2.932E + 6	9.063E + 4	3.639E + 7	1.663E + 9	1.389E + 6	2.638E + 8	6.424E + 9	3.796E + 5	1.668E + 10
F36	AVG	2.425E + 3	**2.415E + 3**	2.582E + 3	2.457E + 3	2.675E + 3	9.097E + 3	2.497E + 3	3.735E + 3	1.021E + 4	2.527E + 3	1.579E + 4
	STD	2.845E + 1	**1.142E + 1**	1.111E + 2	2.446E + 1	4.034E + 1	1.225E + 3	4.891E + 2	2.262E + 2	3.934E + 3	6.469E + 1	8.872E + 2
F37	AVG	**2.600E + 3**	2.674E + 3	1.180E + 4	2.670E + 3	1.161E + 4	6.397E + 4	2.722E + 3	3.401E + 4	6.433E + 4	2.726E + 3	7.101E + 4
	STD	**1.057E-3**	1.105E + 2	3.534E + 3	2.417E + 2	8.967E + 2	2.239E + 3	9.960E + 1	3.029E + 3	4.337E + 3	2.359E + 2	1.331E + 3
F38	AVG	**3.269E + 3**	3.345E + 3	4.438E + 3	3.335E + 3	4.501E + 3	2.319E + 4	3.325E + 3	9.137E + 3	2.372E + 4	3.346E + 3	4.045E + 4
	STD	**3.198E + 1**	5.391E + 1	4.330E + 2	7.690E + 1	3.351E + 2	3.146E + 3	1.004E + 2	1.009E + 3	6.139E + 3	6.533E + 1	6.155E + 3

### Non-parametric test analysis

To comprehensively evaluate the performance of DGRO and validate the experimental results obtained for the CEC2013 and CEC2020 benchmark functions on 30 and 50 dimensions, this section employs two statistical analysis methods: the WRST and the FRT. The WRST is a non-parametric statistical test that independently compares DGRO with other algorithms. A p-value of less than 5% in this test indicates a statistically significant difference between the performance of DGRO and the comparison algorithm^47,48^. Similarly, the FRT, a widely utilized statistical method for comparing multiple algorithms, is applied to rank and analyze the performance of various optimization algorithms across a range of problem instances. This test assigns ranks to algorithms based on their performance scores and calculates the average rank for each algorithm, identifying statistically significant differences in their overall performance^49^. Table 6 presents the experimental results obtained from both the WRST and the FRT for the CEC2013 and CEC2020 functions on 30 and 50 dimensions. The results demonstrate that DGRO consistently achieves a p-value of less than 5% in the WRST when compared to other algorithms, indicating significant differences in its optimization performance. This highlights the effectiveness of DGRO in handling diverse optimization challenges. Furthermore, the FRT results confirm that DGRO consistently ranks as the top-performing algorithm across various dimensions and test functions, reinforcing its robustness and superior capability in solving complex optimization problems.

**Table 6:** Non-Parametric test results.

		DGRO	GRO	GWO	HBA	OBLPFA	PO	RWGWO	SCA	SOA	TBLSBCL	TSO
CEC2013 30 Dim	Wilcoxon P-Value	-	6.313E-3	3.200E-5	7.330E-4	4.000E-6	6.000E-6	1.603E-2	6.000E-6	6.000E-6	1.740E-4	6.000E-6
	Friedman Mean	**1.73**	3	5.5	4.52	7.18	8.68	3.05	7.52	9.61	4.39	10.82
	Friedman Rank	**1**	2	6	5	7	9	3	8	10	4	11
CEC2013 50 Dim	Wilcoxon P-Value	-	7.830E-5	5.606E-6	1.129E-4	4.225E-6	5.606E-6	1.327E-2	5.606E-6	5.606E-6	4.250E-3	5.606E-6
	Friedman Mean	**1.63**	3.36	5.25	4.64	7.13	8.79	2.95	7.61	9.63	4.21	10.82
	Friedman Rank	**1**	3	6	5	7	9	2	8	10	4	11
CEC2020 30 Dim	Wilcoxon P-Value	-	9.097E-2	5.062E-3	2.600E-1	5.062E-3	5.062E-3	1.796E-2	5.062E-3	5.062E-3	7.794E-1	5.062E-3
	Friedman Mean	**2.05**	3.15	6.05	2.6	6.9	9	4.15	8	10	3.1	11
	Friedman Rank	**1**	4	6	2	7	9	5	8	10	3	11
CEC2020 50 Dim	Wilcoxon P-Value	-	9.344E-3	5.062E-3	5.062E-3	5.062E-3	5.062E-3	5.062E-3	5.062E-3	5.062E-3	3.077E-1	5.062E-3
	Friedman Mean	**1.40**	3.55	6.10	2.80	6.90	9.00	4.15	8.00	10.00	3.10	11.00
	Friedman Rank	**1**	4	6	2	7	9	5	8	10	3	11

### Exploration vs. Exploitation and diversity analysis

**Fig. 6:**
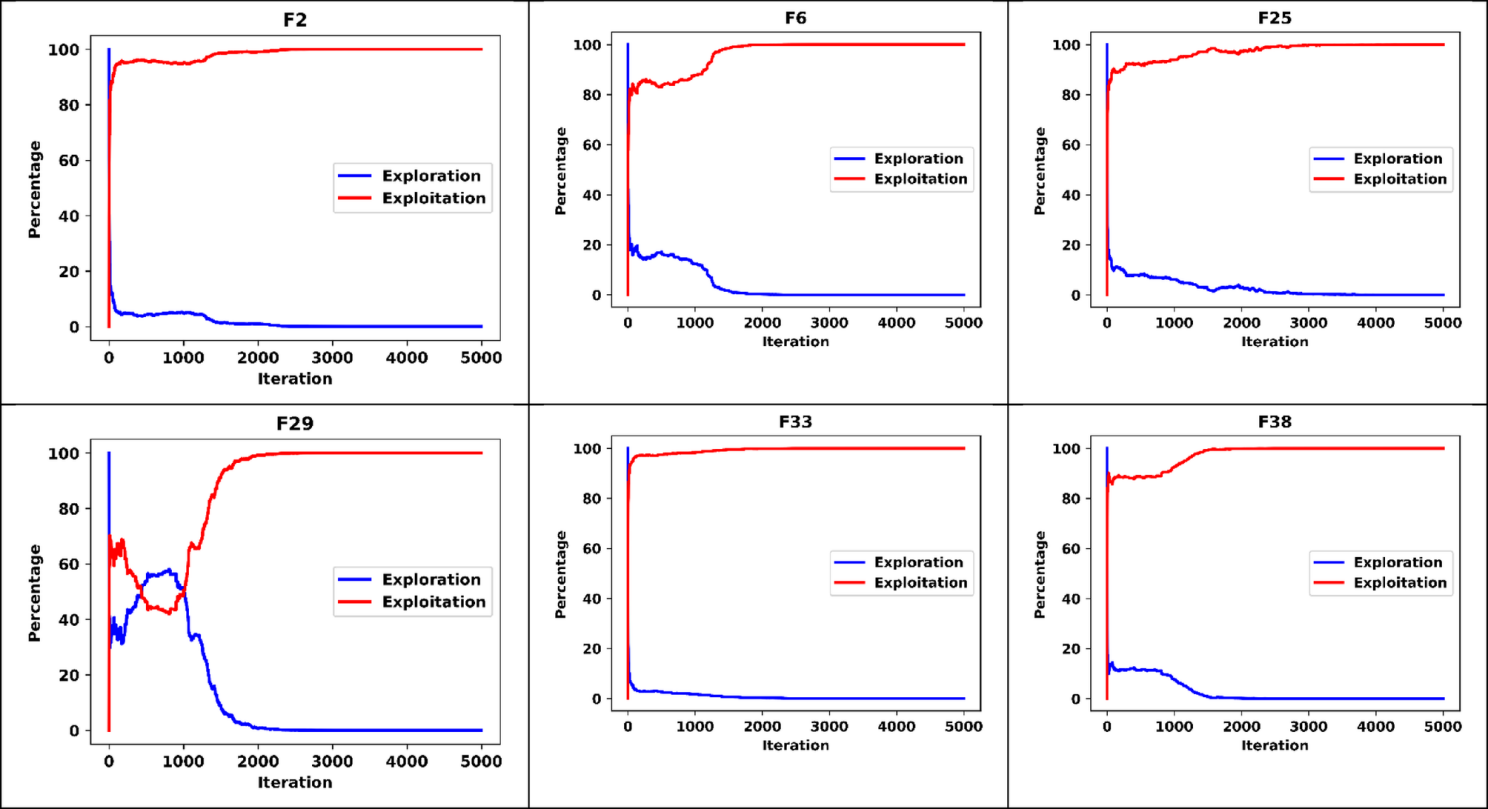
Exploration vs. Exploitation Chart of DGRO on CEC2013 and CEC2020.

**Fig. 7:**
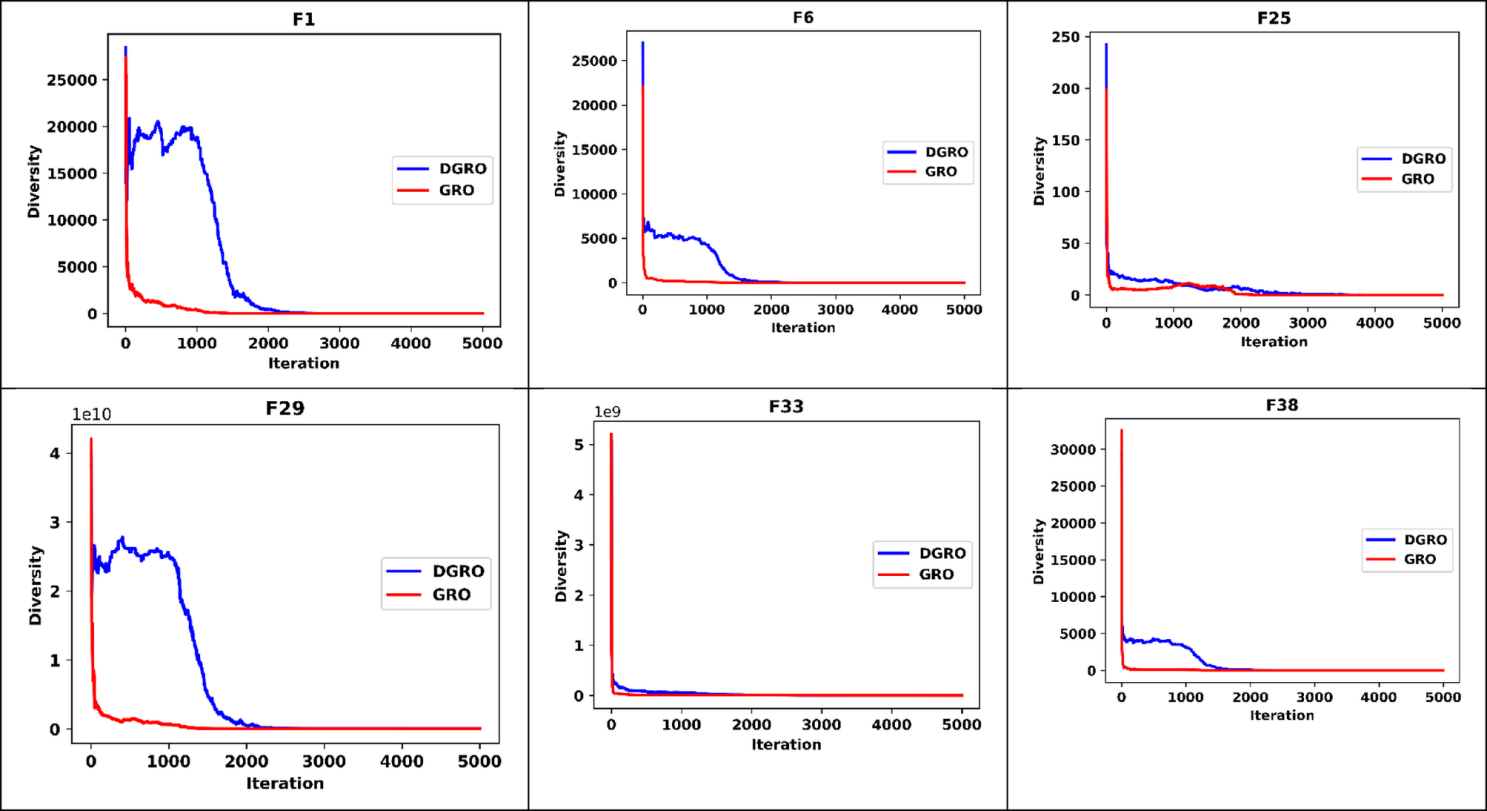
Diversity Chart of DGRO on CEC2013 and CEC2020.

To further validate the performance of DGRO, an extensive analysis was conducted to examine the exploration and exploitation ratios throughout the search process. This analysis involved monitoring the exploration and exploitation patterns exhibited by DGRO during the optimization of the CEC2013 and CEC2020 test functions, as illustrated in Fig. 6. Six representative functions, encompassing both unimodal, multimodal, hybrid, and composite characteristics, were selected to assess DGRO’s capability in balancing exploration and exploitation. The results indicate that DGRO demonstrates a high exploration rate during the initial stages of the search process. However, as the iterations progress, the algorithm dynamically adapts and transitions toward exploitation, effectively converging toward optimal solutions. Additionally, a diversity analysis, depicted in Fig. 7, shows that DGRO maintains a higher population diversity compared to GRO during the initial and intermediate phases of the search. This improved diversity allows DGRO to explore the solution space more thoroughly and avoid premature convergence. As the iterations approach the maximum limit, the diversity gradually decreases, reflecting the algorithm’s focus on exploitation in promising regions of the search space. This behavior highlights DGRO’s ability to balance exploration and exploitation dynamically and efficiently. In conclusion DGRO demonstrates a more balanced and efficient transition between exploration and exploitation phases compared to GRO due to the fusion of SNM and WAM strategies. The algorithm’s dynamic behavior throughout the search process effectively addresses diverse optimization challenges, showcasing its versatility and robustness in handling complex optimization problems. These findings reinforce DGRO’s capability to achieve superior performance and adaptability across various optimization scenarios.

### Computation time

Table 7 presents the average computation times (in seconds) for DGRO and various algorithms on the CEC2013 and CEC2020 benchmarks at 30 and 50 dimensions. For CEC2013 at 30 dimensions, DGRO requires 153.02 s, higher than GRO (75.04 s) but comparable to other algorithms like GWO (136.80 s) and PO (137.81 s). At 50 dimensions, DGRO demonstrates improved scalability with a computation time of 115.89 s, slightly exceeding GRO (109.99 s) but remaining competitive with algorithms like RWGWO (108.16 s). For CEC2020, DGRO records 69.47 s at 30 dimensions and 74.25 s at 50 dimensions, reflecting efficient performance relative to other MAs. Although GRO achieves the lowest computation times (33.64 and 48.47 s), DGRO’s higher computational cost correlates with its superior optimization performance, especially in handling complex functions. Conclusively DGRO balances computational efficiency and effectiveness, offering robust performance with only a moderate increase in runtime due to the advanced exploration and exploitation mechanisms of SNM and WAM. This trade-off highlights DGRO’s scalability and competitiveness in solving diverse optimization problems.

**Table 7:** Average computation Time(seconds).

	DGRO	GRO	GWO	HBA	OBLPFA	PO	RWGWO	SCA	SOA	TBLSBCL	TSO
CEC2013 30 Dim	153.02	**75.04**	136.80	87.16	201.65	137.81	143.65	128.35	86.73	121.54	79.29
CEC2013 50 Dim	115.89	109.99	**102.05**	130.49	123.75	159.30	108.16	161.50	129.80	157.19	121.31
CEC2020 30 Dim	69.47	**33.64**	94.35	46.69	113.62	55.08	97.01	86.77	45.83	81.28	38.52
CEC2020 50 Dim	74.25	**48.47**	89.06	63.99	74.08	64.25	89.40	85.57	62.76	83.22	54.22

### Engineering problem

#### Welded beam design problem

The Welded Beam Design (WBD) is a real world engineering problem, incorporating multiple design variables such as cost minimization under constraints including shear stress (τ), deflection at the end of the beam (δ), bending stress (θ), critical buckling load (*Pc*), and boundary conditions^50^. WBD accounts for four primary design parameters: (₁), (₂), (₃), and (₄)^51^. The main objective of this task is to construct a welded beam at the lowest possible cost.

The WBD problem is expressed as follows in Eqs. (17)-(33):

17 $$\:X=\left[{x}_{1},{x}_{2},{x}_{3},{x}_{4}\right]=[h,l,t,b]$$

18 $$\:f\left(x\right)=1.10471{x}_{1}^{2}{x}_{2}+0.04811{x}_{3}{x}_{4}\left(14.0+{x}_{2}\right)$$

19 $$\:{g}_{1}\left(x\right)=\tau\:\left(x\right)-\text{13,600}\le\:0$$

20 $$\:{g}_{2}\left(x\right)=\sigma\:\left(x\right)-\text{30,000}\le\:0$$

21 $$\:{g}_{3}\left(x\right)={x}_{1}-{x}_{4}\le\:0$$

22 $$\:{g}_{4}\left(x\right)=0.10471{x}_{1}^{2}+0.04811{x}_{3}{x}_{4}\left(14+{x}_{2}\right)-5.0\le\:0$$

23 $$\:{g}_{5}\left(x\right)=0.125-{x}_{1}\le\:0$$

24 $$\:{g}_{6}\left(x\right)=\delta\:\left(x\right)-0.25\le\:0$$

25 $$\:{g}_{7}\left(x\right)=6000-{p}_{c}\left(x\right)\le\:0$$

26 $$\:\tau\:\left(x\right)=\sqrt{{\left({\tau\:}^{{\prime\:}}\right)}^{2}+\left(2\tau\:{\tau\:}^{{\prime\:}}\right)\frac{{x}_{2}}{2R}+{\left({\tau\:}^{{\prime\:}{\prime\:}}\right)}^{2}}$$

27 $$\:{\tau\:}^{{\prime\:}}=\frac{6000}{\sqrt{2}{x}_{1}{x}_{2}},{\tau\:}^{{\prime\:}{\prime\:}}=\frac{MR}{J}$$

28 $$\:M=6000\left(14+\frac{{x}_{2}}{2}\right)$$

29 $$\:R=\sqrt{\frac{{x}_{2}^{2}}{4}+{\left(\frac{{x}_{1}+{x}_{3}}{2}\right)}^{2}}$$

30 $$\:J=2\left\{{x}_{1}{x}_{2}\sqrt{2}\left[\frac{{x}_{2}^{2}}{12}+{\left(\frac{{x}_{1}+{x}_{3}}{2}\right)}^{2}\right]\right\}$$

31 $$\:\sigma\:\left(x\right)=\frac{\text{504,000}}{{x}_{4}{x}_{3}^{2}}$$

32 $$\:\delta\:\left(x\right)=\frac{\text{65,856,000}}{\left(30\cdot\:{10}^{6}\right){x}_{4}{x}_{3}^{3}}$$

33 $$\:\:{p}_{c}\left(x\right)=\frac{4.013\left(30\cdot\:{10}^{6}\right)\sqrt{\frac{{x}_{3}^{2}{x}_{4}^{6}}{36}}}{196}\left(1-\frac{{x}_{3}}{28}\sqrt{\frac{30\cdot\:{10}^{6}}{4\left(12\cdot\:{10}^{6}\right)}}\right)$$

While: $$\:0.1\le\:{x}_{1},{x}_{4}\le\:2\text{\:and\:}0.1\le\:{x}_{2},{x}_{3}\le\:10$$

#### Speed reducer problem

The primary objective of this optimization procedure is to reduce the weight of the speed reducer while complying with the limits imposed by its fundamental components^52^. The important design factors considered are : the gear face width ($\:{x}_{1}$), the teeth module ($\:{x}_{2}$), the number of pinion teeth ($\:{x}_{3}$), the length of the first shaft between bearings ($\:{x}_{4}$), the length of the second shaft between bearings ($\:{x}_{5}$), and the diameters of the first ($\:{x}_{6}$) and second shafts ($\:{x}_{7}$). The mathematical formulation is given in Eq. (34), subject to the constraints in Eqs. (35)–(45).

34 $$\:\begin{aligned}f\left(x\right)&= 0.7854{x}_{1}{x}_{2}^{2}\left(3.3333{x}_{3}^{2}+14.9334{x}_{3}-43.0934\right)\\ &\quad -1.508{x}_{1}\left({x}_{6}^{2}+{x}_{7}^{2}\right)+7.4777\left({x}_{6}^{3}+{x}_{7}^{3}\right)\\ &\quad +0.7854\left({x}_{4}{x}_{6}^{2}+{x}_{5}{x}_{7}^{2}\right)\end{aligned}$$

35 $$\:{g}_{1}\left(x\right)=\left(27/{x}_{1}{x}_{2}^{2}{x}_{3}\right)-1\le\:0$$

36 $$\:{g}_{2}\left(x\right)=\left(397.5/{x}_{1}{x}_{2}^{2}{x}_{3}\right)-1\le\:0$$

37 $$\:{g}_{3}\left(x\right)=\left(1.93{x}_{4}^{3}/{x}_{2}{x}_{3}{x}_{6}^{4}\right)-1\le\:0$$

38 $$\:{g}_{4}\left(x\right)=\left(1.93{x}_{5}^{3}/{x}_{2}{x}_{3}{x}_{7}^{4}\right)-1\le\:0$$

39 $$\:{g}_{5}\left(x\right)=\left(1/110{x}_{6}^{3}\right)\sqrt{{\left(745{x}_{4}/{x}_{2}{x}_{3}\right)}^{2}+16.9\times\:{10}^{6}}-1\le\:0$$

40 $$\:{g}_{6}\left(x\right)=\left(1/85{x}_{7}^{3}\right)\sqrt{{\left(745{x}_{4}/{x}_{2}{x}_{3}\right)}^{2}+157.5\times\:{10}^{6}}-1\le\:0$$

41 $$\:{g}_{7}\left(x\right)=\left({x}_{2}{x}_{3}/40\right)-1\le\:0$$

42 $$\:{g}_{8}\left(x\right)=\left(5{x}_{2}^{2}/{x}_{1}\right)-1\le\:0$$

43 $$\:{g}_{9}\left(x\right)=\left({x}_{1}/12{x}_{2}\right)-1\le\:0$$

44 $$\:{g}_{10}\left(x\right)=\left(\left(1.5{x}_{6}+1.9\right)/{x}_{4}\right)-1\le\:0$$

45 $$\:{g}_{11}\left(x\right)=\left(\left(1.1{x}_{7}+1.9\right)/{x}_{5}\right)-1\le\:0$$

with 2.6 ≤ x_1_ ≤ 3.6, 0.7 ≤ x_2_ ≤ 0.8, 2.6 ≤ x_1_ ≤ 3.6, 17 ≤ x_3_ ≤ 28, 7.3 ≤ x_4_ ≤ 7.8, 7.8 ≤ x_5_ ≤ 8.3, 2.9 ≤ x_6_ ≤ 3.9 and 5 ≤ x_7_ ≤ 5.5.

#### Planar Three-Bar truss design problem

The three-bar planar truss structure is designed to optimally reduce the weight of the structure^53^.With $\:X$ = [$\:{x}_{1}$,$\:\:{x}_{2}$], $\:{x}_{1}$ and $\:{x}_{2\:}$are randomly chosen from the range [0, 1]. The following are the equations for this optimization problem as seen in Eqs. (46)-(49).

Minimize:

46 $$\:\:\:f({x}_{1},{x}_{2})=l\times\:\left(2\sqrt{2}{x}_{1}+{x}_{2}\right)$$

Constrained by:

47 $$\:{G}_{1}=\frac{\sqrt{2}{x}_{1}+{x}_{2}}{\sqrt{2}{x}_{1}2+2{x}_{1}{x}_{2}}P-\sigma\:\le\:0\:$$

48 $$\:{G}_{2}=\frac{{x}_{2}}{\sqrt{2}{x}_{1}2+2{x}_{1}{x}_{2}}P-\sigma\:\le\:0$$

49 $$\:{G}_{3}=\frac{1}{\sqrt{2}{x}_{2}+{x}_{1}}P-\sigma\:\le\:0$$

Where: $$\:l=100\text{c}\text{m};P=\frac{2kN}{{\text{c}\text{m}}^{2}};\sigma\:=\frac{2\text{k}\text{N}}{{\text{c}\text{m}}^{2}}$$

#### Gear train problem

The Gear train Problem is structured to determine the number of teeth needed on each gear in order to realize a target speed ratio between the input and output shafts^54^. In this setup, A, B, C, and D denote the tooth counts of the corresponding gears mounted on each wheel. The objective in the gear train design problem is to minimize the deviation in angular velocity between the input and output shaft, as given in Eq. (50).

50 $$\:\left.\begin{array}{c}\text{m}\text{i}\text{n}\text{f}\left(x\right)={\left(\frac{1}{6.931}-\frac{{x}_{2}{x}_{3}}{{x}_{1}{x}_{4}}\right)}^{2}\\\:\overrightarrow{x}=[A,B,C,D]=\left[{x}_{1},{x}_{2},{x}_{3},{x}_{4}\right],\:12\le\:{x}_{i}\le\:60\end{array}\right\}$$

#### The tubular column design problem

The Tubular Column Design Problem focuses on optimizing the design of a uniform tubular column subjected to compressive loading, with the objective of minimizing cost^55^. This problem involves two design variables: the mean diameter of the column, *d* (₁), and the thickness, *t* (₂). The column is constructed from a material characterized by a yield stress of σ_γ_ = 500 kgf/cm² and a modulus of elasticity of E = 0.85 × 10⁶ kgf/cm². The optimization model for this design task is defined as follows in Eqs. (51)-(59).

Minimize:

51 $$\:f\left(X\right)=9.82{x}_{1}{x}_{2}+2{x}_{1}$$

Subject to:

52 $$\:{g}_{1}\left(X\right)=\frac{2500}{\pi\:{x}_{1}{x}_{2}}-{\sigma\:}_{y}\le\:0$$

53 $$\:{g}_{2}\left(X\right)=\frac{2500}{\pi\:{x}_{1}{x}_{2}}-\frac{{\pi\:}^{2}E\left({x}_{1}^{2}+{x}_{2}^{2}\right)}{8(250{)}^{2}}\le\:0$$

54 $$\:{g}_{3}\left(X\right)=2-{x}_{1}\le\:0$$

55 $$\:{g}_{4}\left(X\right)={x}_{1}-14\le\:0$$

56 $$\:{g}_{5}\left(X\right)=-{x}_{2}+0.2\le\:0$$

57 $$\:{g}_{6}\left(X\right)={x}_{2}-0.8\le\:0$$

Variable Range:

58 $$\:2\le\:{x}_{1}\le\:14$$

59 $$\:0.2\le\:{x}_{2}\le\:0.8$$

#### Piston lever problem

The primary goal is to determine the optimal positions of the piston components *H* (*x*_1_), *B* (*x*_2_), *D* (*x*_3_), and *X* (*x*_4_) by minimizing the oil volume required as the piston lever is raised from 0° to 45°^56^. The objective function for this problem is defined as follows in Eqs. (60)-(68).

Minimize :

60 $$\:f(H,B,D,X)=\frac{1}{4}\pi\:{D}^{2}\left({L}_{2}-{L}_{1}\right)$$

Subject to:

61 $$\:{g}_{1}=QL\text{c}\text{o}\text{s}\theta\:-RF\le\:0\:\text{}$$

62 $$\:{g}_{2}=Q(L-X)-{M}_{\text{m}\text{a}\text{x}}\le\:0$$

63 $$\:{g}_{3}=1.2\left({L}_{2}-{L}_{1}\right)-{L}_{1}\le\:0$$

64 $$\:{g}_{4}=D/2-B\le\:0$$

Where:

65 $$\:R=\frac{|-X(X\text{s}\text{i}\text{n}\theta\:+H)+H(B-X\text{c}\text{o}\text{s}\theta\:\left)\right|}{\sqrt{(X-B{)}^{2}+{H}^{2}}}$$

66 $$\:F=\pi\:P{D}^{2}/4$$

67 $$\:{L}_{1}=\sqrt{(X-B{)}^{2}+{H}^{2}}$$

68 $$\:{L}_{2}={\sqrt{(X\text{s}\text{i}\text{n}45+H{)}^{2}+(B-X\text{c}\text{o}\text{s}45{)}^{2}}}^{2}$$

$$\:\theta\:=45;Q=\text{10,000}\text{l}\text{b}\text{s};M=1.8\times\:{10}^{6}\:\text{l}\text{b}\text{s};P=1500\text{p}\text{s}\text{i};L=240$$

Range: $$\:0.05\le\:x1,x2,x4\le\:500;:0.05\le\:x3\le\:120$$.

#### The corrugated bulkhead problem

The Corrugated Bulkhead Problem focuses on minimizing the weight of a bulkhead in a chemical tanker featuring a corrugated design^55^. The design variables involved include the width (₁), depth (₂), length (₃), and plate thickness (₄) of the bulkhead. The mathematical formulation of this optimization problem is presented as follows in in Eqs. (69)-(75).

69 $$\:f\left(X\right)=\frac{5.885{x}_{4}\left({x}_{1}+{x}_{3}\right)}{{x}_{1}+\sqrt{\left|{x}_{3}^{2}-{x}_{2}^{2}\right|}}$$

Subject to:

70 $$\:{g}_{1}\left(X\right)=-{x}_{4}{x}_{2}\left(0.4{x}_{1}+\frac{{x}_{3}}{6}\right)+8.94\left({x}_{1}+\sqrt{\left|{x}_{3}^{2}-{x}_{2}^{2}\right|}\right)\le\:0$$

71 $$\:{g}_{2}\left(X\right)=-{x}_{4}{x}_{2}^{2}\left(0.2{x}_{1}+\frac{{x}_{3}}{12}\right)+2.2{\left(8.94\left({x}_{1}+\sqrt{\left|{x}_{3}^{2}-{x}_{2}^{2}\right|}\right)\right)}^{4/3}\le\:0$$

72 $$\:{g}_{3}\left(X\right)=-{x}_{4}+0.0156{x}_{1}+0.15\le\:0$$

73 $$\:{g}_{4}\left(X\right)=-{x}_{4}+0.0156{x}_{3}+0.15\le\:0$$

74 $$\:{g}_{5}\left(X\right)=-{x}_{4}+1.05\le\:0$$

75 $$\:{g}_{6}\left(X\right)=-{x}_{3}+{x}_{2}\le\:0$$

Variable Range:

$$\:0\le\:{x}_{1},{x}_{2},{x}_{3}\le\:100$$

$$\:0\le\:{x}_{4}\le\:5$$

The experimental results for the engineering optimization problems highlight the performance of DGRO in comparison to other MAs across various engineering problems. The results, summarized in Table 8, include evaluations of both the AVG and the STD for each algorithm. Details of each engineering problem are provided in Table 9. The experiments were conducted over 5000 iterations, with 30 independent runs. DGRO consistently achieves competitive results across most problems, demonstrating its robustness and effectiveness in handling constrained and high-dimensional engineering problems. For the WBP, DGRO achieves the optimal value of 1.683 similar to other optimizers with a minimal STD, indicating stability and reliability. In the SRD, DGRO’s performance matches other compared algorithms, achieving an average value of 2.987E + 3. Similarly, for the TBTD, DGRO maintains consistent performance with an average 2.639E + 2, demonstrating its efficiency in handling low-dimensional, constrained problems. In the GTD, DGRO exhibits exceptional precision, with an average value of 1.166E-28, reflecting its capability to converge to near optimal solutions in constrained problem, HBA achieved the optimal solution. For the CBHD, DGRO achieved similar solution with several optimizer maintaining optimal values with a low standard deviation, which signifies a strong balance between exploration and exploitation. Similarly, DGRO demonstrates competitive performance in the TCD, achieving an average value of 3.015E + 1, consistent with other algorithms. In the PLD, DGRO achieves reliable results with an average of 1.057, emphasizing its adaptability to complex and constrained design problem. Across all engineering problems, the results indicate that DGRO effectively balances exploration and exploitation, maintains population diversity, and adapts dynamically to varying constraints and dimensions. These characteristics allow DGRO to match or outperform established algorithms, demonstrating its scalability and versatility in solving complex engineering optimization tasks. This reinforces its potential as a reliable and efficient optimization tool in practical engineering applications.

**Table 8:** Experimental results of engineering problem.

		DGRO	GRO	GWO	HBA	OBLPFA	PO	RWGWO	SCA	SOA	TBLSBCL	TSO
WBD	AVG	**1.683**	**1.683**	**1.683**	**1.683**	**1.683**	**1.683**	**1.683**	**1.683**	1.970	**1.683**	2.569
	STD	**0**	1.605E-7	2.806E-4	6.379E-7	1.714E-2	1.829E-2	6.554E-4	7.854E-4	2.755E-1	3.094E-2	1.631E-1
SRD	AVG	**2.987E + 3**	**2.987E + 3**	2.989E + 3	**2.987E + 3**	**2.987E + 3**	**2.987E + 3**	2.988E + 3	2.991E + 3	3.044E + 3	2.990E + 3	2.534E + 5
	STD	**4.547E-13**	3.041E-2	2.837	2.099E-3	4.488	4.054	2.858	2.545	4.576E + 1	1.432E + 1	9.282E + 5
TBTD	AVG	**2.639E + 2**	**2.639E + 2**	**2.639E + 2**	**2.639E + 2**	**2.639E + 2**	**2.639E + 2**	**2.639E + 2**	**2.639E + 2**	2.656E + 2	**2.639E + 2**	2.661E + 2
	STD	**1.621E-6**	1.496E-5	4.543E-4	5.043E-6	1.521E-4	1.895E-4	7.899E-4	8.692E-4	6.044	5.407E-5	5.036
GTD	AVG	1.166E-28	6.347E-26	2.275E-19	**0**	7.701E-25	9.354E-26	1.181E-18	2.087E-18	8.462E-14	4.220E-24	3.881E-7
	STD	1.937E-17	1.064E-20	1.341E-13	1.026E-15	7.595E-22	**4.024E-22**	4.650E-14	4.731E-14	9.022E-10	2.575E-21	5.642E-3
CBD	AVG	**1.340**	**1.340**	**1.340**	**1.340**	**1.340**	**1.340**	**1.340**	**1.340**	1.670	**1.340**	1.499
	STD	2.211E-7	6.481E-7	6.132E-6	3.414E-7	3.295E-6	1.576E-6	1.311E-5	2.423E-5	8.071E-1	**2.208E-7**	1.402E-2
TCD	AVG	**3.015E + 1**	**3.015E + 1**	**3.015E + 1**	**3.015E + 1**	**3.015E + 1**	**3.015E + 1**	**3.015E + 1**	**3.015E + 1**	3.017E + 1	**3.015E + 1**	3.024E + 1
	STD	**1.421E-14**	1.567E-7	3.906E-4	**1.421E-14**	4.986E-7	4.352E-7	3.906E-4	2.914E-4	1.413E-1	3.603E-7	2.324E-1
PLD	AVG	**1.057**	**1.057**	**1.057**	**1.057**	**1.057**	**1.057**	**1.057**	1.058	1.137	1.060	9.281
	STD	**6.655E-14**	1.362E-5	6.203E + 1	4.998E + 1	2.891E-2	2.989E + 1	5.658E + 1	6.207E + 1	1.158	7.179E + 1	1.386E + 3
CBHD	AVG	**6.843**	**6.843**	**6.843**	**6.843**	**6.843**	**6.843**	**6.843**	**6.843**	9.354	**6.843**	7.703
	STD	**4.441E-15**	1.779E-7	7.353E-4	7.180E-11	3.192E-2	3.881E-2	7.530E-4	7.937E-4	3.401	1.778E-2	1.803

**Table 9:** Engineering problem description.

Engineering Problem	Abbreviation	No of constraints	Dimension
Welded Beam Problem	WBP	7	4
Speed Reducer Problem	SRD	11	7
Three Bar Truss Problem	TBTD	3	2
Gear Train Problem	GTD	0	4
Tubular Column Problem	TCD	6	2
Piston Lever Problem	PLD	4	4
Corrugated Bulkhead Problem	CBHD	6	4

## Conclusion

This research presents the DGRO, an enhanced version of the original GRO, designed to address its limitations in exploration and exploitation. DGRO incorporates two innovative mechanisms: the SNM and the WAM. The SNM enhanced exploration through a dynamic stochastic mechanism, which improved population diversity and local optimal avoidance, while enabling a seamless transition from exploration to exploitation. WAM further strengthens exploration by promoting interactions and adaptive learning, resulting in more efficient convergence toward global optima. These modifications significantly improve DGRO’s ability enabling it to achieve robust optimization performance. The effectiveness of DGRO was thoroughly evaluated through extensive experiments on benchmark functions from the CEC2013 and CEC2020 test suites, as well as on seven complex engineering optimization problems. Comparative analysis against various MAs demonstrated DGRO’s superior performance across both low-dimensional (30) and high-dimensional (50) CEC problems. Statistical evaluations using the WRST and FRT further validated DGRO’s effectiveness, consistently ranking it as the most optimal algorithm among the compared methods. These results highlight DGRO’s advancements in addressing GRO’s inherent limitations, firmly establishing it as a robust and competitive optimization framework capable of handling complex, multimodal problems.

Future work offers promising avenues to extend the capabilities of DGRO. Efforts will be focused on enhancing computational efficiency by optimizing the algorithm’s runtime through parallelization techniques, particularly for high-dimensional and computationally intensive problems. Additionally, the algorithm’s scope will be expanded to address multi-objective optimization challenges, broadening its applicability in real-world scenarios. These advancements will further strengthen DGRO as a versatile and powerful optimization tool in both academic research and practical applications.

## Data Availability

The data obtained through the experiments are available upon request from the corresponding author.

## References

[1] Devi, R. et al. IRKO: An Improved Runge-Kutta Optimization Algorithm for Global Optimization Problems, *Computers, Materials and Continua*, vol. 70, pp. 4803–4827, Oct. (2021). 10.32604/cmc.2022.020847

[2] Shi, Y. & Zhang, Y. The neural network methods for solving traveling salesman problem. *Procedia Comput. Sci.* **199**, 681–686. 10.1016/j.procs.2022.01.084 (Jan. 2022).

[3] Gandomi, A. H., Yang, X. S. & Alavi, A. H. Cuckoo search algorithm: a metaheuristic approach to solve structural optimization problems, *Engineering with Computers*, vol. 29, no. 1, pp. 17–35, Jan. (2013). 10.1007/s00366-011-0241-y

[4] Shirazi, A., Ceberio, J. & Lozano, J. A. Spacecraft trajectory optimization: A review of models, objectives, approaches and solutions. *Prog. Aerosp. Sci.* **102**, 76–98. 10.1016/j.paerosci.2018.07.007 (Oct. 2018).

[5] Afroz, Z., Shafiullah, G. M., Urmee, T., Shoeb, M. A. & Higgins, G. Predictive modelling and optimization of HVAC systems using neural network and particle swarm optimization algorithm, *Building and Environment*, vol. 209, p. 108681, Feb. (2022). 10.1016/j.buildenv.2021.108681

[6] Afroz, Z., Shafiullah, G., Urmee, T., Shoeb, M. & Higgins, G. Predictive modelling and optimization of HVAC systems using neural network and particle swarm optimization algorithm, *Building and Environment*, vol. 209, p. 108681, Dec. (2021). 10.1016/j.buildenv.2021.108681

[7] Deng, L. & Liu, S. Advancing photovoltaic system design: an enhanced social learning swarm optimizer with guaranteed stability. *Comput. Ind.* **164**, 104209. 10.1016/j.compind.2024.104209 (Jan. 2025).

[8] Wei, F., Wu, Y., Xu, S. & Wang, X. Accurate visible light positioning technique using extreme learning machine and meta-heuristic algorithm. *Opt. Commun.* **532**, 129245. 10.1016/j.optcom.2022.129245 (Apr. 2023).

[9] Rodriguez-Molins, M., Salido, M. A. & Barber, F. A GRASP-based metaheuristic for the Berth Allocation Problem and the Quay Crane Assignment Problem by managing vessel cargo holds, *Appl Intell*, vol. 40, no. 2, pp. 273–290, Mar. (2014). 10.1007/s10489-013-0462-4

[10] Gharehchopogh, F. S. Quantum-inspired metaheuristic algorithms: comprehensive survey and classification. *Artif. Intell. Rev.* **56** (6), 5479–5543. 10.1007/s10462-022-10280-8 (Jun. 2023).

[11] Yang, W., Xia, K., Li, T., Xie, M. & Zhao, Y. An Improved Transient Search Optimization with Neighborhood Dimensional Learning for Global Optimization Problems, *Symmetry*, vol. 13, no. 2, Art. no. 2, Feb. (2021). 10.3390/sym13020244

[12] Li, S., Gong, W. & Gu, Q. A comprehensive survey on meta-heuristic algorithms for parameter extraction of photovoltaic models. *Renew. Sustain. Energy Rev.* **141**, 110828. 10.1016/j.rser.2021.110828 (May 2021).

[13] Wang, L., Cao, Q., Zhang, Z., Mirjalili, S. & Zhao, W. Artificial rabbits optimization: A new bio-inspired meta-heuristic algorithm for solving engineering optimization problems. *Eng. Appl. Artif. Intell.* **114**, 105082. 10.1016/j.engappai.2022.105082 (Sep. 2022).

[14] Azizi, M., Talatahari, S. & Gandomi, A. H. Fire Hawk optimizer: a novel metaheuristic algorithm. *Artif. Intell. Rev.* **56** (1), 287–363. 10.1007/s10462-022-10173-w (Jan. 2023).

[15] Hashim, F. A., Houssein, E. H., Hussain, K., Mabrouk, M. S. & Al-Atabany, W. Honey Badger algorithm: new metaheuristic algorithm for solving optimization problems. *Math. Comput. Simul.* **192**, 84–110. 10.1016/j.matcom.2021.08.013 (Feb. 2022).

[16] Deng, L. & Liu, S. Snow ablation optimizer: A novel metaheuristic technique for numerical optimization and engineering design. *Expert Syst. Appl.* **225**, 120069. 10.1016/j.eswa.2023.120069 (Sep. 2023).

[17] Pan, J. S., Zhang, L. G., Wang, R. B., Snášel, V. & Chu, S. C. Gannet optimization algorithm : A new metaheuristic algorithm for solving engineering optimization problems, *Mathematics and Computers in Simulation*, vol. 202, pp. 343–373, Dec. (2022). 10.1016/j.matcom.2022.06.007

[18] Zhao, S., Zhang, T., Ma, S. & Chen, M. Dandelion optimizer: A nature-inspired metaheuristic algorithm for engineering applications. *Eng. Appl. Artif. Intell.* **114**, 105075. 10.1016/j.engappai.2022.105075 (Sep. 2022).

[19] Sörensen, K. Metaheuristics—the metaphor exposed. *Int. Trans. Oper. Res.* **22** (1), 3–18. 10.1111/itor.12001 (2015).

[20] Abualigah, L. et al. Improved reptile search algorithm by salp swarm algorithm for medical image segmentation. *J. Bionic Eng.* **20** (4), 1766–1790. 10.1007/s42235-023-00332-2 (Jul. 2023). 10.1007/s42235-023-00332-2 PMC9902839 36777369

[21] Deng, L. & Liu, S. An enhanced slime mould algorithm based on adaptive grouping technique for global optimization. *Expert Syst. Appl.* **222**, 119877. 10.1016/j.eswa.2023.119877 (Jul. 2023).

[22] Song, H., Bei, J., Zhang, H., Wang, J. & Zhang, P. Hybrid algorithm of differential evolution and flower pollination for global optimization problems. *Expert Syst. Appl.* **237**, 121402. 10.1016/j.eswa.2023.121402 (Mar. 2024).

[23] Song, Y., Wang, F. & Chen, X. An improved genetic algorithm for numerical function optimization. *Appl. Intell.* **49** (5), 1880–1902. 10.1007/s10489-018-1370-4 (May 2019).

[24] He, Y. & Wang, M. An improved chaos sparrow search algorithm for UAV path planning. *Sci. Rep.* **14** (1), 366. 10.1038/s41598-023-50484-8 (Jan. 2024). 10.1038/s41598-023-50484-8 PMC10764786 38172279

[25] Deng, L. & Liu, S. A Novel Hybrid Grasshopper Optimization Algorithm for Numerical and Engineering Optimization Problems, *Neural Process. Lett.*, vol. 55, no. 7, pp. 9851–9905, Mar. (2023). 10.1007/s11063-023-11230-3

[26] Ye, M., Zhou, H., Yang, H., Hu, B. & Wang, X. Multi-Strategy Improved Dung Beetle Optimization Algorithm and Its Applications, *Biomimetics*, vol. 9, no. 5, Art. no. 5, May (2024). 10.3390/biomimetics9050291 10.3390/biomimetics9050291 PMC11117942 38786501

[27] Deng, L. & Liu, S. Incorporating Q-learning and gradient search scheme into JAYA algorithm for global optimization, *Artif. Intell. Rev.*, vol. 56, no. Suppl 3, pp. 3705–3748, Oct. (2023). 10.1007/s10462-023-10613-1

[28] Zolfi, K. Gold rush optimizer. A new population-based metaheuristic algorithm. *Oper. Res. Decisions*. **33** (1), 113–150. 10.37190/ord230108 (2023).

[29] Saglam, M., Bektas, Y. & Karaman, O. A. Dandelion optimizer and gold rush optimizer Algorithm-Based optimization of multilevel inverters. *Arab. J. Sci. Eng.* **49** (5), 7029–7052. 10.1007/s13369-023-08654-3 (May 2024).

[30] Khajuria, R., Sharma, P., Lamba, R., Kumar, R. & Raju, S. Model Parameter Extraction of Solar PV Cell Using Gold Rush Optimizer, in *Advances in Clean Energy and Sustainability, Volume 2*, S. S. V. Tatiparti and S. Seethamraju, Eds., Singapore: Springer Nature, pp. 163–173. (2024). 10.1007/978-981-97-5419-9_15

[31] Chen, J., Shen, Q. & Zhu, S. Gray wolf optimizer based on gold rush optimizer, in *International Conference on Automation Control, Algorithm, and Intelligent Bionics (ACAIB 2024)*, SPIE, Sep. pp. 1070–1074. (2024). 10.1117/12.3039342

[32] Lyu, L., Kong, G., Yang, F., Li, L. & He, J. Augmented gold rush optimizer is used for engineering optimization design problems and UAV path planning. *IEEE Access.* **12**, 134304–134339. 10.1109/ACCESS.2024.3445269 (2024).

[33] Kong, W. et al. Wireless sensor network coverage optimization based on novel multi-strategy gold rush optimizer. *Electron. Lett.* **60** (11), e13249. 10.1049/ell2.13249 (Jun. 2024).

[34] Nie, Y., Liu, Y., Zhao, W. & Li, J. Optimizing Linear Antenna Arrays with The Chaotic Gold Rush Optimizer, in *IEEE 14th International Conference on Electronics Information and Emergency* Communication *(ICEIEC)*, May 2024, pp. 31–34. (2024). 10.1109/ICEIEC61773.2024.10561817

[35] Mirjalili, S. et al. Salp Swarm Algorithm: A bio-inspired optimizer for engineering design problems, *Advances in Engineering Software*, vol. 114, pp. 163–191, Dec. (2017). 10.1016/j.advengsoft.2017.07.002

[36] Salgotra, R. & Singh, U. The naked mole-rat algorithm. *Neural Comput. Applic*. **31** (12), 8837–8857. 10.1007/s00521-019-04464-7 (Dec. 2019).

[37] Lang, J. J., Qu, B. Y., Suganthan, P. N. & Hernández-Díaz, A. G. Problem Definitions and Evaluation Criteria for the CEC 2013 Special Session on Real-Parameter Optimization, Computational Intelligence Laboratory, Zhengzhou University, Zhengzhou China And Technical Report, Nanyang Technological University, Singapore, Technical Report Technical Report 201212, Spring 2013.

[38] Wagdy, A. et al. Problem Definitions and Evaluation Criteria for the CEC 2021 Special Session and Competition on Single Objective Bound Constrained Numerical Optimization, Nanyang Technological University, Singapore, Singapore, Technical Report, Nov. (2020).

[39] Mirjalili, S., Mirjalili, S. M. & Lewis, A. Grey Wolf optimizer. *Adv. Eng. Softw.* **69**, 46–61. 10.1016/j.advengsoft.2013.12.007 (Mar. 2014).

[40] Lian, J. et al. Apr., Parrot optimizer: Algorithm and applications to medical problems, *Computers in Biology and Medicine*, vol. 172, p. 108064, (2024). 10.1016/j.compbiomed.2024.108064 10.1016/j.compbiomed.2024.108064 38452469

[41] Mirjalili, S. SCA: A sine cosine algorithm for solving optimization problems. *Knowl. Based Syst.* **96**, 120–133. 10.1016/j.knosys.2015.12.022 (Mar. 2016).

[42] Dhiman, G. & Kumar, V. Seagull optimization algorithm: Theory and its applications for large-scale industrial engineering problems, *Knowledge-Based Systems*, vol. 165, pp. 169–196, Feb. (2019). 10.1016/j.knosys.2018.11.024

[43] Qais, M. H., Hasanien, H. M. & Alghuwainem, S. Transient search optimization: a new meta-heuristic optimization algorithm, *Appl Intell*, vol. 50, no. 11, pp. 3926–3941, Nov. (2020). 10.1007/s10489-020-01727-y

[44] Talha, A., Bouayad, A. & Malki, M. O. C. An improved pathfinder algorithm using opposition-based learning for tasks scheduling in cloud environment. *J. Comput. Sci.* **64**, 101873. 10.1016/j.jocs.2022.101873 (Oct. 2022).

[45] Gupta, S. & Deep, K. A novel random walk grey Wolf optimizer. *Swarm Evol. Comput.* **44**, 101–112. 10.1016/j.swevo.2018.01.001 (Feb. 2019).

[46] Qaraad, M., Amjad, S., Hussein, N. K. & Elhosseini, M. A. Addressing constrained engineering problems and feature selection with a time-based leadership salp-based algorithm with competitive learning, *Journal of Computational Design and Engineering*, vol. 9, no. 6, pp. 2235–2270, Dec. (2022). 10.1093/jcde/qwac095

[47] Adegboye, O. R., Deniz, E. & Ülker Hybrid artificial electric field employing cuckoo search algorithm with refraction learning for engineering optimization problems, *Sci Rep*, vol. 13, no. 1, Art. no. 1, Mar. (2023). 10.1038/s41598-023-31081-1 10.1038/s41598-023-31081-1 PMC10008842 36907914

[48] García, S., Molina, D., Lozano, M. & Herrera, F. A study on the use of non-parametric tests for analyzing the evolutionary algorithms’ behaviour: a case study on the CEC’2005 special session on real parameter optimization. *J. Heuristics*. **15** (6), 617. 10.1007/s10732-008-9080-4 (May 2008).

[49] García, S., Fernández, A., Luengo, J. & Herrera, F. Advanced nonparametric tests for multiple comparisons in the design of experiments in computational intelligence and data mining: experimental analysis of power. *Inf. Sci.* **180** (10), 2044–2064. 10.1016/j.ins.2009.12.010 (May 2010).

[50] Kamil, A. T., Saleh, H. M. & Abd-Alla, I. H. A Multi-Swarm structure for particle swarm optimization: solving the welded beam design problem. *J. Phys. : Conf. Ser.* **1804** (1), 012012. 10.1088/1742-6596/1804/1/012012 (Feb. 2021).

[51] Deng, L. & Liu, S. A multi-strategy improved slime mould algorithm for global optimization and engineering design problems. *Comput. Methods Appl. Mech. Eng.* **404**, 115764. 10.1016/j.cma.2022.115764 (Feb. 2023).

[52] Alabdulhafith, M. et al. A modified Bonobo optimizer with its application in solving engineering design problems. *IEEE Access.* **12**, 134948–134984. 10.1109/ACCESS.2024.3455550 (2024).

[53] Qaraad, M. et al. Comparing SSALEO as a scalable large scale global optimization algorithm to High-Performance algorithms for Real-World constrained optimization benchmark. *IEEE Access.* **10**, 95658–95700. 10.1109/ACCESS.2022.3202894 (2022).

[54] Duankhan, P., Sunat, K., Chiewchanwattana, S. & Nasa-Ngium, P. The Differentiated Creative search (DCS): Leveraging Differentiated knowledge-acquisition and Creative realism to address complex optimization problems. *Expert Syst. Appl.* **252**, 123734. 10.1016/j.eswa.2024.123734 (2024).

[55] Şenol, M. E., Çetin, T. & Turan, M. E. Performance Comparison of Recent Metaheuristic Algorithms on Engineering Design Optimization Problems, *Bitlis Eren Üniversitesi Fen Bilimleri Dergisi*, vol. 13, no. 4, Art. no. 4, Dec. (2024). 10.17798/bitlisfen.1514951

[56] Seyyedabbasi, A. & Kiani, F. Sand Cat swarm optimization: a nature-inspired algorithm to solve global optimization problems. *Eng. Comput.* **39** (4), 2627–2651. 10.1007/s00366-022-01604-x (Aug. 2023).

